# Error-Gated Hebbian Rule: A Local Learning Rule for Principal and Independent Component Analysis

**DOI:** 10.1038/s41598-018-20082-0

**Published:** 2018-01-30

**Authors:** Takuya Isomura, Taro Toyoizumi

**Affiliations:** Laboratory for Neural Computation and Adaptation, RIKEN Brain Science Institute, 2-1 Hirosawa, Wako, Saitama 351-0198 Japan

## Abstract

We developed a biologically plausible unsupervised learning algorithm, *error-gated Hebbian rule* (*EGHR*)*-β*, that performs principal component analysis (PCA) and independent component analysis (ICA) in a single-layer feedforward neural network. If parameter *β* = 1, it can extract the subspace that major principal components span similarly to Oja’s subspace rule for PCA. If *β* = 0, it can separate independent sources similarly to Bell-Sejnowski’s ICA rule but without requiring the same number of input and output neurons. Unlike these engineering rules, the EGHR-*β* can be easily implemented in a biological or neuromorphic circuit because it only uses local information available at each synapse. We analytically and numerically demonstrate the reliability of the EGHR-*β* in extracting and separating major sources given high-dimensional input. By adjusting *β*, the EGHR-*β* can extract sources that are missed by the conventional engineering approach that first applies PCA and then ICA. Namely, the proposed rule can successfully extract hidden natural images even in the presence of dominant or non-Gaussian noise components. The results highlight the reliability and utility of the EGHR-*β* for large-scale parallel computation of PCA and ICA and its future implementation in a neuromorphic hardware.

## Introduction

The ability to separate blind sources (blind source separation; BSS)^1,2^ is important for animals to perceive their environment. However, the most basic form of Hebbian plasticity, where synaptic strengths are updated by the pure product of pre- and postsynaptic activity, is insufficient to perform BSS and a state-dependent Hebbian plasticity is a strong candidate mechanism for neuronal BSS^3^. A biologically plausible independent component analysis (ICA) algorithm called the error-gated Hebbian rule (EGHR) was recently developed^4^. The EGHR modulates the magnitude of Hebbian plasticity by a global (error) factor. This global factor represents average activity of output neurons, which can be easily computed and read out in a biological system. This is the so-called *local learning rule* to achieve ICA. By contrast, engineering ICA rules^5–7^ are difficult to implement using neural networks (the so-called non-local learning rules^8^) because each neuron needs non-physiological information such as synaptic strengths between other neurons. Mathematical and numerical analyses of the EGHR support the stability of ICA solutions and the absence of major spurious solutions. Unlike some other ICA rules, this is the case even when the number of neurons is greater than that of the sources (the undercomplete condition)^4^. Thus, the EGHR is a biologically plausible and reliable unsupervised learning rule for ICA.

Apart from ICA, principal component analysis (PCA) is another classic method widely used for data compression^9^, i.e., removing minor components and extracting principal components from a high-dimensional dataset. PCA is often used to explore the low-dimensional hidden representation underlying the data. The brain is also believed to perform PCA-like learning. For example, visual inputs are largely high dimensional; thus, the visual system needs to reduce these dimensions in order to perceive objects^10^. However, similarly to ICA algorithms, current PCA algorithms are either non-local^11^ or requires a specialized circuit that subtracts a leading principal component one by one in a sequential manner^12^. A simple local learning rule would be useful to explore neuronal mechanisms underlying the PCA-like learning.

Here, we develop a new local learning rule called EGHR-*β*. It smoothly interpolates between performing PCA and ICA as parameter *β* that controls the weight of PCA varies. This algorithm can achieve dimensionality reduction and ICA simultaneously. While PCA is often used as a pre-processing step before applying ICA to perform BSS, this cascade is not always optimal. Notably, depending on parameter *β*, the EGHR-*β* can extract sources with large and negative kurtosis (i.e., sub-Gaussian sources) that the PCA-to-ICA cascade cannot extract in the presence of large noise. Hence, the EGHR-*β* can solve BSS by separately extracting either major or sub-Gaussian independent sources from the ensemble of high-dimensional sensory inputs.

In the following sections, we first analytically and numerically show that depending on *β*, the EGHR-*β* can extract either principal components or sub-Gaussian sources from high-dimensional inputs. Next, more generally, we demonstrate that the EGHR-*β* can extract the hidden natural images by removing noise. Finally, the advantages and limitations of the EGHR-*β* are discussed.

## Results

### A novel local learning rule for PCA and ICA (the EGHR-*β*)

**Figure 1:**
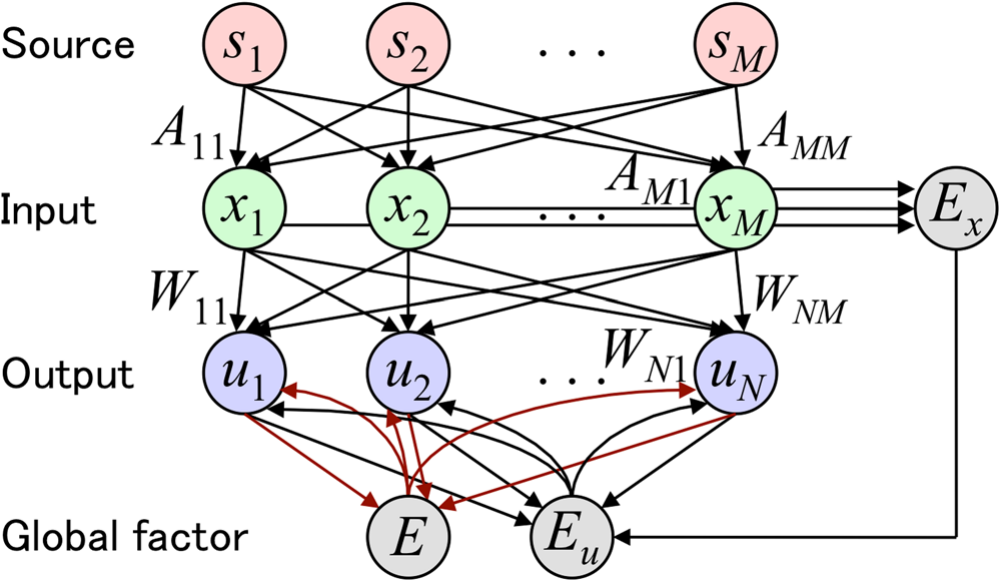
Model structure of EGHR-*β*. Note that *s*_1_, …, *s*_*M*_ are hidden sources; *x*_1_, …, *x*_*M*_ are sensory inputs; *u*_1_, …, *u*_*N*_ a re neural outputs; *A*_11_, …, *A*_1*M*_, *A*_21_, …, *A*_*MM*_ are elements of a mixing matrix; *W*_11_, …, *W*_1*M*_, *W*_21_, …, *W*_*NM*_ are synaptic strengths; and scalars *E*, *E*_*u*_, and *E*_*x*_ are global factors.

First, we define a novel, biologically plausible local learning rule that performs BSS by combining PCA and ICA, termed as the EGHR-*β*. Let us consider a BSS problem of inverting a linear generative model using a single-layer feedforward neural network. The generative model consists of the *M*-dimensional hidden sources ***s*** ≡ (*s*_1_, …, *s*_*M*_)^*T*^ that are independently generated from source distributions *p*(***s***) ≡ Π_*i*_*p*_*i*_(*s*_*i*_) and the *M*-dimensional sensory inputs **x** ≡ (*x*_1_, …, *x*_*M*_)^*T*^ ≡ *A***s** that are generated by multiplying the sources with an *M* × *M* mixing matrix *A*. A single-layer neural network receives sensory inputs **x** and computes the *N*-dimensional neural outputs u ≡ (*u*_1_, …, *u*_*N*_)^*T*^ ≡ *W***x** by multiplying the inputs with an *N* × *M* synaptic strength matrix *W*, where *N* ≤ *M* (see also Fig. 1). The cost function of the EGHR-*β* is defined by

1 $$L\equiv \mathrm{(1}-\beta )\mathop{\underbrace{\langle \frac{1}{2}{(E({\bf{u}})-\langle E({\bf{u}})\rangle )}^{2}-E({\bf{u}})\rangle }}\limits_{{\rm{ICA}}\,{\rm{term}}}+\beta \mathop{\underbrace{\langle \frac{1}{2}{({E}_{u}({\bf{u}})-{E}_{x}({\bf{x}}))}^{2}\rangle }}\limits_{{\rm{PCA}}\,{\rm{term}}},$$

where global error signals *E*(**u**), *E*_*u*_(**u**), and *E*_*x*_(**x**) are respectively defined by

2 $$\begin{array}{ll}E({\bf{u}}) & \equiv -\mathrm{log}\,{p}_{0}({\bf{u}}),\\ {E}_{u}({\bf{u}}) & \equiv \,\frac{1}{2}(|{\bf{u}}{|}^{2}-\langle |{\bf{u}}{|}^{2}\rangle ),\\ {E}_{x}({\bf{x}}) & \equiv \,\frac{1}{2}(|{\bf{x}}{|}^{2}-\langle |{\bf{x}}{|}^{2}\rangle \mathrm{).}\end{array}$$

As shown later, the first and second terms of *L* represent the cost for ICA and PCA, respectively. Note that 0 ≤ *β* ≤ 1 is a parameter that controls the weight of PCA, 〈•〉 is an expectation over *p*(**x**), and *p*_0_(•) is the prior distribution that the sources are assumed to follow. Hence, a gradient descent learning rule of synaptic weights that minimizes *L* is given by

3 $$\begin{array}{ll} & [{\rm{E}}GHR-\beta ]:\\  & \quad \dot{W}\propto -\frac{\partial L}{\partial W}=-\langle \mathrm{(1}-\beta )\mathop{\underbrace{(E({\bf{u}})-{E}_{0})g({\bf{u}}){{\bf{x}}}^{T}}}\limits_{{\rm{ICA}}\,{\rm{term}}}+\beta \mathop{\underbrace{({E}_{u}({\bf{u}})-{E}_{x}({\bf{x}})){\bf{u}}{{\bf{x}}}^{T}}}\limits_{{\rm{PCA}}\,{\rm{term}}}\rangle ,\end{array}$$

where the dot over *W* denotes a temporal derivative, *g*(**u**) ≡ d*E*(**u**)/d**u** is a nonlinear activation function, and *E*_0_ ≡ 1 + 〈*E*(**u**)〉 = 1 − 〈*logp*_0_(**u**)〉. We will refer to Eq. (3) as the EGHR-*β*. Note that this definition of *E*_0_ is slightly different from the original definition 1 − 〈log*p*_0_(**s**)〉^4^ but the resulting behavior turns out to be quite similar (see below for comparison). In the following, *E*(**u**), *E*_*u*_(**u**), and *E*_*x*_(**x**) are referred to as global factors (global signals) that represent neuron non-specific error signals. The cost function of the EHGR-*β* consists of the ICA and PCA terms weighted by 1 − *β* and *β*, respectively, and its derivative provides a local learning rule for PCA and ICA. As we will see, the ICA term (the first term of Eq. (3)) makes the outputs independent of each other, while the PCA term (the second term) increases the correlation between the output and input squared-norms by decreasing (*E*_*u*_(**u**) − *E*_*x*_(**x**)) close to zero. Importantly, the EGHR-*β* can be represented using only local connections because *W* is updated according to the product of pre- and post-neurons’ activities and the global signal (Fig. 1). This property is highly desirable for parallel computing and neuromorphic engineering (see Discussion). The EGHR-*β* becomes a local learning rule for ICA when *β* = 0 and that for PCA when *β* = 1. More generally, it can extract principal components from high-dimensional inputs while separating signals into individual sources when 0 < *β* < 1.

### The features of EGHR-*β*

 We start by investigating how the PCA and ICA terms of the EGHR-*β* are related to previously proposed non-local learning rules: Oja’s subspace rule for PCA^11^ and Bell-Sejnowski’s ICA rule^5,6^, respectively.

A simple analysis shows that the PCA term of the EGHR-*β* is equal to Oja’s subspace rule for PCA^11^ up to a multiplication with a positive definite matrix when the sources independently follow Gaussian distributions (see Eq. (8) in Methods). Next, the ICA term of the EGHR-*β* is equivalent to Bell-Sejnowski’s ICA rule around the neighborhood of ICA solutions when the number of input and output neurons are equal (*M* = *N*) and the source distribution is given by *p*(**s**) ∝ Π_*i*_exp(−*b*|*s*_*i*_|^*a*^) with positive constants *a* and *b* (see Eqs (12)–(13) in Methods). Note that the definition of *E*_0_ in this paper is slightly altered from the original one^4^ to straightforwardly demonstrate the relationship with Bell-Sejnowski’s rule. However, the resulting ICA performance is similar to the original version–mathematical analyses give the same linear stability condition for ICA solutions (see Methods and Supplementary Information); and numerical simulations show the absence of major spurious solutions when random mixing matrices with up to 20 dimensional sources are studied (Fig. S1) and the robustness of the outcome to the choice of nonlinear function *g*(**u**), derived within the sub- or super-Gaussian family (Fig. S2).

Unlike the classical learning rules, the EGHR-*β* can perform these computations only using local information available at each synapse. Moreover, unlike Bell-Sejnowski’s rule, its ICA term can handle a greater number of inputs than the number of output neurons, which makes the EGHR-*β* a great candidate to perform both dimensionality reduction and separation of independent sources. Notably, beyond the above conditions, the behavior of the EGHR-*β* can be better than Oja’s subspace rule and/or Bell-Sejnowski’s ICA rule as we analytically and numerically study in the following. Throughout the result section, we use a uniform prior distribution (*p*_0_(*s*_*i*_) = $1/2\sqrt{3}$ for |*s*_*i*_| < $\sqrt{3}$ or 0 for otherwise) to preferentially extract sub-Gaussian sources with negative kurtosis.

**Figure 2:**
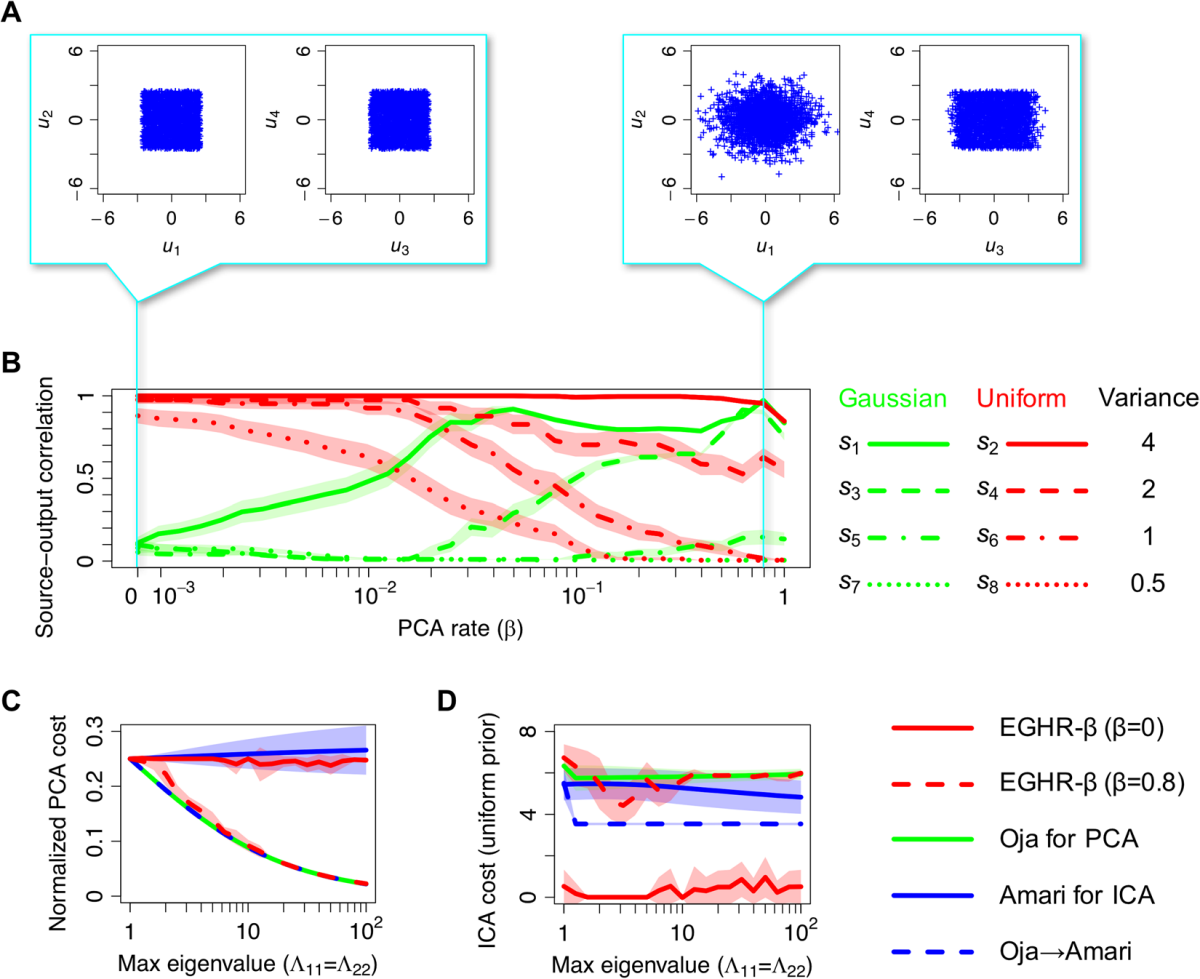
Results of EGHR-*β*. (**A**) Final distribution of outputs **u** = (*u*_1_, *u*_2_, *u*_3_, *u*_4_)^*T*^ with PCA rate *β* = 0 (left panels) and *β* = 0.8 (right panels). Panels show samples of output signals pooled over 10^4^ step displayed in *u*_1_ − *u*_2_ and *u*_3_ − *u*_4_ planes. When *β* = 0, final states of **u** represent sources that follow a uniform distribution, while when *β* = 0.8, they represent major components (top four). (**B**) Correlations between outputs and sources depending on PCA rate *β*. Horizontal axis is PCA rate 0 ≤ *β* ≤ 1, while vertical axis is value of correlation between specific source and output that best describes the source, ${\rm{\arg }}\,{\max }_{i}|{\rm{corr}}({u}_{i},{s}_{j})|$. Green curves represent correlations with Gaussian sources (*s*_1_, *s*_3_, *s*_5_, *s*_7_), while red curves represent correlations with uniform sources (*s*_2_, *s*_4_, *s*_6_, *s*_8_). Eigenvalues of the mixing matrix *A* (i.e., variances of sources) are defined as Λ_11_ = Λ_22_ = 4, Λ_33_ = Λ_44_ = 2, Λ_55_ = Λ_66_ = 1, and Λ_77_ = Λ_88_ = 0.5. Simulations are conducted 40 times for each parameter set, and mean is shown. Shaded areas represent standard error. (**C**), (**D**) Performance of EGHR-*β* (with *β* = 0 and 0.8) is compared with that of other three rules: Oja’s subspace rule for PCA^11^, Amari’s ICA rule^7^, and cascade of Oja and Amari rules. Maximum eigenvalue (horizontal axis) indicates amplitude of largest sources (Λ_11_ = Λ_22_), while other eigenvalues are defined such that ${{\rm{\Lambda }}}_{33}={{\rm{\Lambda }}}_{44}={{\rm{\Lambda }}}_{11}^{\mathrm{2/3}},{{\rm{\Lambda }}}_{55}={{\rm{\Lambda }}}_{66}={{\rm{\Lambda }}}_{11}^{\mathrm{1/3}}$, and Λ_77_ = Λ_88_ = 1. In (**C**), PCA performance is evaluated by the normalized PCA cost (Eq. (6) in Methods divided by the sum of all eigenvalues of *A*), while in (**D**), ICA performance is evaluated by the ICA cost (Eq. (9) in Methods) assuming a uniform prior distribution. Simulations are conducted 10 times for each parameter set, and mean is shown. Shaded areas represent standard deviation. See Methods for detail on experimental parameters. Note that source codes of EGHR-*β* are appended as Supplementary Source Codes.

We analytically study the existence and stability of the solutions of the EGHR-*β* (see Eqs (14)–(18) in Methods for details) and find that the EGHR-*β* can perform PCA without assuming Gaussian sources and ICA without assuming the equal number of input and output neurons. Namely, (1) if *β* ≈ 1, the only stable fixed point of the EGHR-*β* is such that the outputs are spanned by the major principal components; hence, the EGHR-*β* with *β* ≈ 1 performs PCA (see Case 1 in Methods and Supplementary Methods S2, S3); and (2) if *β* ≈ 0, the only stable fixed point is such that the outputs represent sub-Gaussian independent sources; hence, the EGHR-*β* with *β* ≈ 0 performs ICA (see Case 2 in Methods and S2, S3). These properties are also confirmed by numerical simulations, where four independent Gaussian sources and four independent uniformly-distributed sources with different variances are mixed as inputs (Fig. 2). Typical outputs with *β* = 0 and 0.8 are illustrated in Fig. 2A. When *β* = 0.8, the EGHR-*β* succeeded in extracting the subspace of four major principal components from eight-dimensional data (PCA-like condition), while when *β* = 0, the EGHR-*β* succeeded in extracting sub-Gaussian sources (ICA-like condition). Note that we showed the result of *β* = 0.8 here (rather than *β* = 1) because, in addition to performing PCA, the EGHR-*β* can separate independent sub-Gaussian sources. The preference of sources gradually shifts from the ICA-like to the PCA-like one as *β* increases (Fig. 2B).

For comparison with the EGHR-*β*, we consider three non-local algorithms: Oja’s subspace rule for PCA^11^, Amari’s ICA rule^7^, and the cascade of the Oja and Amari rules (see Eqs (5) and (11) in Methods). Note that the results of Bell-Sejnowski’s ICA rule^5,6^ are the same as those of Amari’s ICA rule. PCA^13^ and ICA^7^ cost functions are used as measures (see also Eqs (6) and (9) in Methods for their details), and plotted as the spread of eigenvalues is continuously changed. As expected from the mathematical analyses (see Methods and Supplementary Methods S2–S4), both the EGHR-*β* (*β* = 0.8) and Oja’s subspace rule can extract a subspace of major principal components by reducing the normalized PCA cost by a similar amount (Fig. 2C). Note that the Oja’s subspace rule achieves the theoretical optimum. Next, we explore how these different methods reduce the ICA cost that assumes a uniform prior distribution *p*_0_ (Fig. 2D). This cost function is minimized if independent uniform sources are extracted as outputs. Interestingly, reducing this cost function is not trivial for conventional learning rules. Amari’s ICA rule alone cannot separate sources as it works only when the number of neurons matches that of unknown sources^4^. A common strategy in this scenario is to first apply PCA and then apply ICA to its output. Interestingly, this PCA-to-ICA cascade fails to reduce the cost function because the first PCA step discards the minor uniformly-distributed sources. Only the EGHR-*β* (*β* = 0) can separately extract minor sub-Gaussian independent sources (Fig. 2D).

### An application to extract natural and artificial images

**Figure 3:**
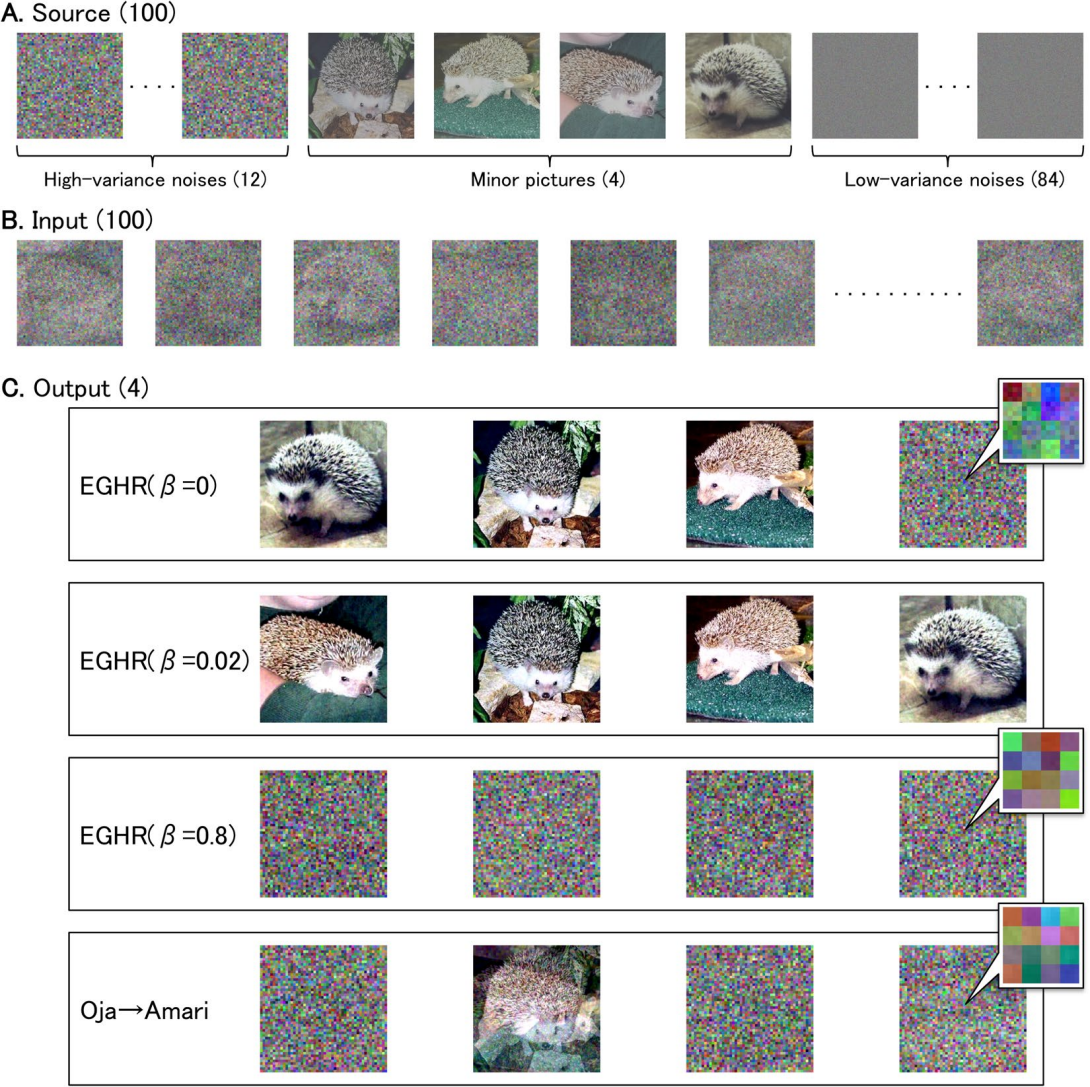
Dimensionality reduction and BSS using natural and artificial images. (**A**) Original natural and noise images as hidden sources. They consist of 12 high-variance colored noise images (var = 0.023, kurt = 0), four minor natural images (hedgehogs; var ≈ 0.02, kurt ≈ −1), and 84 low-variance white noise images (var = 0.002, kurt = −1.2) (var; variance, kurt; kurtosis). (**B**) One hundred randomly superposed images provided as input to neural network. (**C**) Final states of the four-dimensional outputs of neural network reconstructed some original images. Transitions of outputs are shown in Supplementary Movie S1. Top: EGHR-*β* with *β* = 0 extracts and separates three natural images and a mixture of noise images. Second line: EGHR-*β* with *β* = 0.02 successfully extracts and separates all four natural images. Third line: EGHR-*β* with *β* = 0.8 extracts colored noise images (major principal components). Bottom: cascade of Oja and Amari rules extracts mixtures of colored noise and natural images as some natural images correlate with each other and produce a major principal component. Three inset panels in the right display magnified images, which show that only the result of the EHGR-*β* with *β* = 0, but not the others, includes low-variance white noise images. We retrieved these hedgehog pictures from the Caltech101 dataset^40^ and processed them accordingly. See Methods for detail on experimental parameters.

We demonstrate the performance of the EGHR-*β* using mixtures of natural and artificial images as inputs. Twelve high-variance colored noise images with zero kurtosis, four pictures of a distinct hedgehog with negative kurtosis, and 84 low-variance white noise images with negative kurtosis were used as sources (Fig. 3A). The color intensities of the individual pixels were converted to real numbers and then centered to be zero mean following^4,14^ (see also Methods). The 100 images were superposed to produce 100 mixed images using a random but fixed 100 × 100 rotation matrix (Fig. 3B). One pixel was randomly sampled from the identical position of these 100 mixed images at a time, and fed as input into a one-layer feed-forward neural network that has four output neurons (as in Fig. 1). Synaptic strength matrix *W* of the model was updated according to the EGHR-*β* with *β* = 0, 0.02, or 0.8 (Eq. (3)). Note that we used the uniform distribution as the prior *p*_0_ because the natural images tended to follow a sub-Gaussian distribution with negative kurtosis^4^. For comparison, we introduced the same input into a two-layer feed-forward neural network (100-4-4), in which the first and second layers are updated by Oja’s subspace rule^11^ and Amari’s ICA rule^7^, respectively (the cascade of the Oja and Amari rules).

Pictures reconstructed from neural outputs after training are displayed in Fig. 3C (see also Supplementary Movie S1 for the learning process). We found that the cascade of the Oja and Amari rules (Fig. 3C bottom) extracted mixtures of colored noise images and natural images. These images were extracted because ICA rules generally cannot separate mixed Gaussian sources and the mixed hedgehog image represents the primary principal component of the input owing to the small but non-negligible correlation between the four hedgehog images. Hence, the Oja rule extracted the subspace spanned by the three colored noise images and the mixed hedgehog image as major components, and the following Amari rule simply segregated this non-Gaussian hedgehog image from the rest. Next, the EGHR-*β* with *β* = 0.8 extracted four high-variance colored noise components (Fig. 3C third line). The reason that the EGHR-*β* dropped the primary principal component (the mixed hedgehog image in Fig. 3C bottom) can be understood from the stability analysis (see Eq. (16) in Methods for details), which shows that if eigenvalues are similar to each other, the EGHR-*β* solution for *β* ≈ 1 becomes more stable when the outputs extract sources with positive large kurtosis. Accordingly, the EGHR-*β* with *β* = 0.8 extracted Gaussian colored noise images rather than the (barely) primary sub-Gaussian principal component (i.e., the mixed hedgehog image). By contrast, the EGHR-*β* with *β* = 0.02 successfully extracted and separated all minor hedgehog images even in the presence of large Gaussian noise (Fig. 3C second line). This *β* = 0.02 parameter preferentially extracted images with negative kurtosis, while discarding low-variance noise. Finally, the result of EGHR-*β* with *β* = 0 varied depending on the initial synaptic weights as it does not efficiently utilize the variance of images. It tended to extract some minor hedgehog images and some mixtures of noise images. Figure 3C top shows an example, where three hedgehog images and one mixed noise image are extracted. Because the *β* = 0 parameter preferentially extracts independent components with negative kurtosis, the extracted noise image included the low-variance sub-Gaussian noise but the algorithm was tolerant to its contamination with colored noise images (see top right inset panel in Fig. 3C for the magnified image). Therefore, the EGHR-*β* can flexibly extract either high-variance images or minor natural images with large and negative kurtosis depending on the tuning of *β*, purely in an unsupervised manner. Furthermore, only the EGHR-*β* with *β* slightly larger than 0, but not the cascade of PCA and ICA algorithms, can extract sources with intermediate variance and negative kurtosis, discarding both high-variance Gaussian noise and low-variance sub-Gaussian noise. This result demonstrates the benefit of performing both PCA and ICA by the same set of neurons.

## Discussion

In this study, we developed a novel learning rule for PCA and ICA, the EGHR-*β*. The EGHR-*β* can compress data by removing minor components and extracting either principal components or sub-Gaussian sources from a high-dimensional dataset by adjusting the parameter *β*. The learning rule updates each synaptic strength in a single-layer linear feedforward network based on the sum of PCA and ICA terms, where each term is given by a simple product of pre- and postsynaptic neurons’ activity and a global scalar factor. Hence, the proposed scheme is much simpler than conventional ICA methods that require non-local information^5–7,15^, dense and plastic lateral inhibition between output neurons^16–18^, or an additional preprocessing stage for PCA to remove background noises^11,19^. This simplicity is a great advantage for the EGHR-*β* because it can reduce the number of processing layers and connections, and the related energy costs, making its implementation in a neuromorphic chip^20^ significantly easier.

If sources follow a Gaussian distribution, we showed that the EGHR-*β* can extract the subspace that principal components span in a way that is mathematically equivalent to the well-known Oja’s subspace rule (see Eq. (8) in Methods). Whereas, if sources follow non-Gaussian distributions, the fixed point and the linear stability are influenced by the kurtosis of discarded components. Because of this property, the EGHR-*β* can robustly perform BSS even in the presence of large Gaussian noise, where a standard cascade of PCA-to-ICA processing cannot. While the EGHR-*β* generally consists of a sum of PCA and ICA terms, we can approximately express it by a single-term three-factor rule when the source distributions are close to Gaussian. In this case, the postsynaptic factor, *g*(**u**), of the ICA term becomes identical to that of the PCA term, **u**, and, hence, the net global error signal becomes the weighted sum of those for the PCA and ICA terms. Note that an additional mechanism may be required to extract minor sources with positive kurtosis (i.e., super-Gaussian sources) because a solution that extracts super-Gaussian sources can be unstable in the presence of large noise.

In biological neural networks, associative (Hebbian) plasticity occurs depending on the timing of pre- and post-neurons’ activity (i.e., a two-factor learning rule)^21–23^. However, recent studies show that third factors, such as neuromodulators^24–29^, GABAergic inputs^30,31^, and glial factors^32^, can modulate the original associative plasticity in various ways (the so-called three-factor learning rule^33,34^). The EGHR-*β* is one of the three-factor learning rules and each of its PCA and ICA terms updates the synaptic strength by the product of pre- and postsynaptic activities and a global error signal. The global error signals are defined as the non-linear sum of output activities, similarly to inhibitory neurons in the visual cortex^35,36^, and they change the learning rate and even invert Hebbian to anti-Hebbian in a manner similar to what has been reported for GABA^31^. Note that the PCA and ICA learning could happen at non-overlapping timing in a biological setup, such as in a wake and sleep condition^37^. Importantly, this process only uses information that actual neurons can access via their synaptic connections to achieve PCA and ICA. Thus, the EGHR-*β* is a local rule, while conventional methods, such as the Oja and Amari rules^7,11^, use non-local information (synaptic strengths of non-connected neurons) to update synaptic strengths. This demonstrates the utility of the EGHR-*β* also as a model of learning processes in a biological neural network.

In summary, we developed the EGHR-*β* by enhancing the original EHGR to handle largely high-dimensional inputs in a biological manner. The EGHR-*β* would be useful in engineering for improving object recognition accuracy in noisy background. Because the EGHR-*β* is easily implemented with recently advanced neuromorphic chips and can process the “big data” in parallel with energy efficiency, the EGHR-*β* is expected to have an impact in various fields such as engineering and life science.

## Methods

First, we describe the relationship between the EGHR-*β* and the original EGHR^4^. Next, for comparison with the EHGR-*β*, we introduce non-local PCA^11,13^ and ICA^5–7^ rules. Finally, we analyze fixed points and their linear stability of the EHGR-*β*.

### Relationship between the EGHR-*β* and the original EGHR

In this paper, the definition of the cost function of the ICA part of the EHGR-*β* is slightly different from that of the original EGHR^4^. Their relationship is represented by

4 $$\begin{array}{ll} & \frac{1}{2}\langle {(E({\bf{u}})-\langle E({\bf{s}})\rangle -\mathrm{1)}}^{2}\rangle \\  & \quad \quad \quad \quad =\,\langle \frac{1}{2}{(E({\bf{u}})-\langle E({\bf{u}})\rangle )}^{2}-E({\bf{u}})\rangle +\frac{1}{2}{(\langle E({\bf{u}})\rangle -\langle E({\bf{s}})\rangle )}^{2}+{\rm{const}}\mathrm{.,}\end{array}$$

where the left hand side is the cost of the original EGHR, while the first term in the right is the cost of the ICA part of the EHGR-*β*. The constant factor makes no difference. The second term in the right gives an additional stability to the original EGHR by minimizing the difference between 〈*E*(**u**)〉 and 〈*E*(**s**)〉. However, since the first term of the right hand side (i.e., the ICA part of the EGHR-*β*) alone has the ICA ability, this second term is not necessary (see Supplementary Figures S1 and S2). Moreover, their linear stability conditions around ICA solutions are the same. Although only the original EGHR has an additional tr(*dK*)^2^ term in its second-derivative^4^, this does not change the linear stability condition. Indeed, the second-derivative of the ICA part of the EGHR-*β* is more similar to that of the well-known Bell-Sejnowski’s ICA rule^5,6^ around ICA solutions as we describe below.

### Conventional non-local rules for comparison

In this section, we introduce conventional learning rules to perform either PCA or ICA. Unlike the EGHR-*β* introduced above, all rules introduced here are non-local. For a comparison of PCA, Oja’s subspace rule for PCA is considered^11^.

5 $$\dot{W}\propto \langle {\bf{u}}({{\bf{x}}}^{T}-{{\bf{u}}}^{T}W)\rangle \mathrm{.}$$

This rule is an enhancement of Oja’s original model^38^ and can extract a subspace that the first to the *N*th principal components span by the *N*-dimensional neural output. Importantly, Oja’s subspace rule is a non-local rule because it needs to calculate the product of *W*^*T*^ and **u** (alternatively, it needs to prepare new neurons ***y*** = *W*^*T*^**u**, but how to extract *W*^*T*^ in a biological setting is open to discussion). While Oja’s subspace rule does not have a cost function, Xu proposed a similar learning rule that is derived as a gradient descent rule of a cost function and achieves PCA^13^. The cost function is defined by

6 $${L}_{X}\equiv \frac{1}{2}\langle {|{\bf{x}}-{W}^{T}{\bf{u}}|}^{2}\rangle $$

because the purpose of PCA is to obtain a representation using a small number of output units with the least loss. The dynamics of *W* are defined by

7 $$\dot{W}\propto -\frac{\partial {L}_{X}}{\partial W}=\langle {\bf{u}}({{\bf{x}}}^{T}-{{\bf{u}}}^{T}W)+({\bf{u}}-W{W}^{T}{\bf{u}}){{\bf{x}}}^{T}\rangle \mathrm{.}$$

Equation (7) is termed the least mean squared error-based PCA^13^. Empirically, the second term converges to zero quickly. Consequently, the least mean squared error-based PCA finds the same solution as Oja’s subspace rule (Eq. (5)). We use this cost function in Fig. 2 to quantify the success of PCA.

Indeed, when sources follow a unit Gaussian distribution, the PCA term of the EGHR-*β* becomes Oja’s subspace rule^11^ except a multiplication with a positive definite matrix. Suppose *β* = 1 and **x** follow a Gaussian distribution with zero mean and variance of *AA*^*T*^. From Bussgang theorem^39^, the EGHR-*β* becomes

8 $$\begin{array}{ll}\frac{\partial L}{\partial W} & =\,\frac{1}{2}\langle (|{\bf{u}}{|}^{2}-\langle |{\bf{u}}{|}^{2}\rangle -|{\bf{x}}{|}^{2}+\langle |{\bf{x}}{|}^{2}\rangle ){\bf{u}}{{\bf{x}}}^{T}\rangle \\  & =\,\langle {\bf{u}}{({W}^{T}{\bf{u}}-{\bf{x}})}^{T}+(|{\bf{u}}{|}^{2}-\langle |{\bf{u}}{|}^{2}\rangle -|{\bf{x}}{|}^{2}+\langle |{\bf{x}}{|}^{2}\rangle )I\rangle \langle {\bf{x}}{{\bf{x}}}^{T}\rangle \\  & =\,\langle {\bf{u}}({{\bf{u}}}^{T}W-{{\bf{x}}}^{T})\rangle A{A}^{T}\mathrm{.}\end{array}$$

This is equivalent to Oja’s subspace rule up to a multiplication with positive definite matrix *AA*^*T*^. For a comparison with non-Gaussian sources, see the fixed point analysis of Case 1 below, where their fixed points are also similar.

In addition, for a comparison of BSS ability, Amari’s ICA rule is considered^7^. The cost function of Amari’s ICA rule is defined by the Kullback-Leibler divergence^9^ between *p*(**u**) and *p*_0_(**u**).

9 $${L}_{A}\equiv {D}_{KL}[p({\bf{u}})||{p}_{0}({\bf{u}})]\equiv \langle \mathrm{log}\,p({\bf{u}})-\,\mathrm{log}\,{p}_{0}({\bf{u}})\rangle \mathrm{.}$$

The gradient of *L*_*A*_ gives Bell-Sejnowski’s non-local ICA rule^5,6^

10 $$\dot{W}\propto -\frac{\partial {L}_{A}}{\partial W}={W}^{-T}-\langle g({\bf{u}}){{\bf{x}}}^{T}\rangle ,$$

while the natural gradient of *L*_*A*_ gives Amari’s non-local ICA rule^7^

11 $$\dot{W}\propto -\frac{\partial {L}_{A}}{\partial W}{W}^{T}W=W-\langle g({\bf{u}}){{\bf{u}}}^{T}\rangle W\mathrm{.}$$

The ICA term of the EGHR-*β* is close to Bell-Sejnowski’s ICA rule^5,6^. Suppose *M* = *N*, *β* = 0, and **u** = *K***s** with square matrix *K* ≡ *WA*. From Lemma S1.1 in Supplementary Methods S1, the EGHR-*β* becomes

12 $$\begin{array}{rcl}\frac{\partial L}{\partial K} & = & \langle (E({\bf{u}})-\langle E({\bf{u}})\rangle -\mathrm{1)}\mathop{\underbrace{g({\bf{u}})}}\limits_{Kg({\bf{s}})+dg}{{\bf{s}}}^{T}\rangle \\  & = & K\langle {K}^{T}g({\bf{u}}){{\bf{s}}}^{T}+(\mathop{\underbrace{E({\bf{u}})-\langle E({\bf{u}})\rangle }}\limits_{0}-\mathrm{1)}I\rangle +\langle (E({\bf{u}})-\langle E({\bf{u}})\rangle -\mathrm{1)}dg{{\bf{s}}}^{T}\rangle \\  & = & K{K}^{T}(\langle g({\bf{u}}){{\bf{s}}}^{T}\rangle -{K}^{-T})+\langle (E({\bf{u}})-\langle E({\bf{u}})\rangle -\mathrm{1)}dg{{\bf{s}}}^{T}\rangle \\  & = & K{K}^{T}\frac{\partial {L}_{A}}{\partial K}+\langle (E({\bf{u}})-\langle E({\bf{u}})\rangle -\mathrm{1)}dg{{\bf{s}}}^{T}\rangle ,\end{array}$$

where *dg* ≡ *g*(**u**) − *Kg*(**s**). We numerically check that the second term in the last line is smaller than the first term. Furthermore, when *W* is around ICA solutions (i.e., *K* = *I* + *dK* is close to the identity matrix), from Lemmas S1.1 and S1.3, the EGHR-*β* becomes

13 $$\begin{array}{rcl}\frac{\partial L}{\partial K} & = & (I+dK+d{K}^{T})(\langle g({\bf{u}}){{\bf{s}}}^{T}\rangle -{K}^{-T})+\langle (E({\bf{s}})-\langle E({\bf{s}})\rangle -\mathrm{1)}dg{{\bf{s}}}^{T}\rangle +{\mathscr{O}}(d{K}^{2})\\  & = & (\langle g({\bf{u}}){{\bf{s}}}^{T}\rangle -{K}^{-T})+(dK+d{K}^{T})\mathop{\underbrace{(\langle g({\bf{s}}){{\bf{s}}}^{T}\rangle -I)}}\limits_{0}+{\rm{\Delta }}{\rm{\Omega }}\circ dK+{\mathscr{O}}(d{K}^{2})\\  & = & \frac{\partial {L}_{A}}{\partial K}+{\rm{\Delta }}{\rm{\Omega }}\circ dK+{\mathscr{O}}(d{K}^{2}),\end{array}$$

where *dg* = Diag[*g*′(**s**)]*dK***s** − *dKg*(**s**), ° is Hadamard product (element-wise product), and ΔΩ is a constant matrix that expresses the difference between coefficient matrices for the EGHR-*β* and Bell-Sejnowski’s ICA rule. Specifically, when sources follow *p*(**s**) ∝ Π_*i*_exp(−*b*|*s*_*i*_|^*a*^) with positive constants *a*, *b*, ΔΩ becomes zero. See^4^ for derivation details.

### Fixed point of the EGHR-*β*

Below, we mathematically analyze the fixed points of the EGHR-*β* (Eq. (3)). Suppose mixing matrix *A* consists of *A* = *R*Λ^1/2^*B*. Without loss of generality, *R* and $B\in {{\mathbb{R}}}^{M\times M}$ are rotation matrices, and ${\rm{\Lambda }}\in {{\mathbb{R}}}^{M\times M}$ is a diagonal matrix. Note that the diagonal elements of Λ are the eigenvalues of *AA*^*T*^ up to permutations. Moreover, suppose that *s*_1_, …, *s*_*M*_ independently follow even distributions with zero mean and unit variance. We define a matrix $K\equiv WA\in {{\mathbb{R}}}^{N\times M}$. We investigate fixed points in the following three cases. See Supplementary Methods S2 for derivation details, and the next section for their linear stability analysis.

#### **Case 1**.

Suppose *B* = *I*. If we use the Gaussian prior distribution ${p}_{0}({\bf{u}})={\mathscr{N}}({\bf{u}})$ for the ICA term, where ${\mathscr{N}}(\bullet )$ is a unit Gaussian distribution, the necessary and sufficient condition for a (nonzero) fixed point is

14 $$K=(P,O),$$

where $P\in {{\mathbb{R}}}^{N\times N}$ is a full-rank orthogonal matrix that holds

15 $${P}^{T}P={\rm{Diag}}[\beta {{\rm{\Lambda }}}_{ii}+\frac{1-\beta }{1+{\kappa }_{i}\mathrm{/2}}]\mathrm{.}$$

Note that Diag[•] is a diagonal matrix in which the *i*th (*i* = 1, …, *N*) diagonal element is •, and ${\kappa }_{i}=\langle {s}_{i}^{4}\rangle -3$ is the kurtosis of the *i*th source distribution. If *β* = 1, $P=C{{\rm{\Lambda }}}_{1}^{\mathrm{1/2}}$ satisfies Eq. (15), where $C\in {{\mathbb{R}}}^{N\times N}$ is any rotation matrix and ${{\rm{\Lambda }}}_{1}={\rm{Diag}}[{{\rm{\Lambda }}}_{ii}]\in {{\mathbb{R}}}^{N\times N}$ is any sub-diagonal matrix of Λ. Similarly, a necessary and sufficient condition for a fixed point of Oja’s subspace rule is *K* = (*P*, *O*) with ${P}^{T}P={\tilde{{\rm{\Lambda }}}}_{1}$, where ${\tilde{{\rm{\Lambda }}}}_{1}$ is another *N* × *N*-dimensional sub-diagonal matrix of Λ (see Supplementary Methods S4). Thus, both the outputs of the EGHR-*β* and Oja’s subspace rule span an arbitrary subspace of *N* principal components at a fixed point.

#### **Case 2**.

(A special case of following Case 3) Suppose *β* = 0. Moreover, suppose *s*_1_, …, *s*_*N*_ independently follow the identical even prior distribution *p*_0_(*s*_*i*_) with zero mean and unit variance, and *s*_*N* + 1_, …, *s*_*M*_ independently follow distributions with zero mean and unit variance. Then, $K=(I,O)\in {{\mathbb{R}}}^{N\times M}$ with the *N* × *N* identity matrix *I* and the *N* × (*M* − *N*) zero matrix *O* is a fixed point of the EHGR-*β*. At this fixed point, the outputs represent the *N* independent sources whose distributions are matched to the prior distribution.

#### **Case 3**.

Suppose *β*(≥0) is a small constant, *s*_1_, …, *s*_*N*_ independently follow the identical even distribution *p*_0_(*s*_*i*_) with zero mean and unit variance, and *s*_*N* + 1_, …, *s*_*M*_ independently follow distributions with zero mean and unit variance. Then, $K=(I,O)+{\mathscr{O}}(\beta )\in {{\mathbb{R}}}^{N\times M}$ is a fixed point. See Supplementary Methods S2 for detailed values of ${\mathscr{O}}(\beta )$.

### Linear stability of the EGHR-*β*

Below, we investigate the linear stability of the fixed points described in the above section (Cases 1–3 below are the same cases as those in the above section). See Supplementary Methods S3 for derivation details.

#### **Case 1**.

The fixed point of Eqs (14–15) is linearly stable if and only if

16 $$\beta ({{\rm{\Lambda }}}_{ii}-\mathrm{(1}+{\kappa }_{j}\mathrm{/2)}{{\rm{\Lambda }}}_{jj})+\mathrm{(1}-\beta )(\frac{1}{1+{\kappa }_{i}\mathrm{/2}}-1) > 0\quad {\rm{for}}\,\,1\le i\le N,N+1\le j\le M\mathrm{.}$$

In the special case of *β* = 1 and *s*_*N* + 1_, …, *s*_*M*_ following a unit Gaussian distribution, the condition for linear stability is Λ_*ii*_ ≥ Λ_*jj*_ for 1 ≤ *i* ≤ *N* and *N* + 1 ≤ *j* ≤ *M*. Thus, the state is stable when the output **u** represents a space that is spanned by the first to *N*th principal components, while the state is unstable when **u** involves other minor components, meaning that the EGHR-*β* can extract major principal components. More generally, when *s*_*N* + 1_, …, *s*_*M*_ follow non-Gaussian distributions, the linear stability condition also depends on the kurtosis (*κ*_*i*_ ≥ −2) as shown above.

#### **Case 2**.

The fixed point in Case 2 in the above section is stable if and only if

17 $$\begin{array}{ll}1+{{\rm{\Omega }}}_{ii} > 0\quad  & {\rm{for}}\,\,1\le i=j\le N,\,\,\,\,\,\\ {{\rm{\Omega }}}_{ij}{{\rm{\Omega }}}_{ji} > 1\quad  & {\rm{for}}\,\,1\le i\ne j\le N,\\ {{\rm{\Omega }}}_{ij} > 0\quad  & {\rm{for}}\,\,1\le i\le N,N+1\le j\le M,\end{array}$$

where Ω_*ij*_ is defined by $${{\rm{\Omega }}}_{ii}={\rm{cov}}(-\mathrm{log}\,{p}_{0}({s}_{i}),g^{\prime} ({s}_{i}){s}_{i}^{2})$$ for *i* = *j*, and $${{\rm{\Omega }}}_{ij}={\rm{cov}}(-\mathrm{log}\,{p}_{0}({s}_{i}),g^{\prime} ({s}_{i}))+$$$${\rm{cov}}(-\mathrm{log}\,{p}_{0}({s}_{j}),{s}_{j}^{2})\langle g^{\prime} ({s}_{i})\rangle {\rm{\Theta }}[j\le N]$$ for *i* ≠ *j*. (Note that Θ[*j* ≤ *N*] is 1 for *j* ≤ *N*, and 0 otherwise.) To see how the shape of the source distribution influences the linear stability, let us consider the special case in which *s*_1_, …, *s*_*N*_ follow *p*_0_(*s*_*i*_) ∝ exp(−*b*|*s*_*i*_|^*a*^), where *a* > 0 is a positive constant and *b* > 0 is tuned such that $$\langle {s}_{i}^{2}\rangle =1$$, and *s*_*N* + 1_, …, *s*_*M*_ follow distributions with zero mean and unit variance. In this case, we can straightforwardly show that *a* > 2 is a necessary and sufficient condition to be linearly stable. Namely, when *s*_1_, …, *s*_*N*_ follow a sub-Gaussian distribution (*a* > 2) and *s*_*N* + 1_, …, *s*_*M*_ follow Gaussian or super-Gaussian distributions, *s*_1_, …, *s*_*N*_ are chosen as outputs. By contrast, when *s*_1_, …, *s*_*N*_ follow a super-Gaussian distribution (*a* < 2), *s*_1_, …, *s*_*N*_ may not be extracted simultaneously. Hence, the EGHR-*β* extracts sub-Gaussian sources.

#### **Case 3**.

If we suppose *B* = *I* and *s*_1_, …, *s*_*N*_ follow *p*_0_(*s*_*i*_) ∝ exp(−*b*|*s*_*i*_|^*a*^), the fixed point in Case 3 in the above section is stable if and only if

18 $$\begin{array}{l}\beta \mathrm{(1}+{\kappa }_{i}\mathrm{/2)(}{{\rm{\Lambda }}}_{ii}-\mathrm{1)}\langle g^{\prime} ({s}_{i})\rangle +\mathrm{(1}-\beta )\frac{a-2}{a}\langle g^{\prime} ({s}_{i})\rangle +\beta \mathrm{(1}-\mathrm{(1}+{\kappa }_{j}\mathrm{/2)}{{\rm{\Lambda }}}_{jj}) > 0\\ {\rm{for}}\,\,1\le i\le N,N+1\le j\le M\mathrm{.}\end{array}$$

Hence, the EGHR-*β* can extract either sub-Gaussian or major sources depending on *β*.

For Fig. 2: In the simulations, *M* = dim(**s**) = dim(**x**) = 8 and *N* = dim(**u**) = 4 are used. An 8 × 8 mixing matrix *A* = *R*Λ^1/2^ consists of a rotation matrix *R* and a diagonal matrix of eigenvalues Λ. We suppose that amplitudes of sources satisfy Λ_11_ = Λ_22_ = 4, Λ_33_ = Λ_44_ = 2, Λ_55_ = Λ_66_ = 1, and Λ_77_ = Λ_88_ = 0.5 in Fig. 2A and B, or ${{\rm{\Lambda }}}_{11}={{\rm{\Lambda }}}_{22},{{\rm{\Lambda }}}_{33}={{\rm{\Lambda }}}_{44}={{\rm{\Lambda }}}_{11}^{\mathrm{2/3}},{{\rm{\Lambda }}}_{55}={{\rm{\Lambda }}}_{66}={{\rm{\Lambda }}}_{11}^{\mathrm{1/3}}$, and Λ_77_ = Λ_88_ = 1 in Fig. 2C and D. Moreover, we suppose that odd-numbered sources (*s*_1_, *s*_3_, *s*_5_, *s*_7_) follow a unit Gaussian distribution ($p({s}_{i})={\mathscr{N}}({s}_{i})=\exp (-{s}_{i}^{2}\mathrm{/2)/}\sqrt{2\pi }$), while even-numbered sources (*s*_2_, *s*_4_, *s*_6_, *s*_8_) follow a unit uniform distribution $p({s}_{i})=1/2\sqrt{3}$ for $|{s}_{i}| < \sqrt{3}$ and 0 otherwise). The training time and the learning rate are defined by *T* = 2 × 10^7^ and *η* = 8 × 10^−6^, respectively. In all cases, *R* is a random rotation matrix, and *W* starts from a random matrix in which each element *W*_*ij*_ follows a Gaussian distribution with zero mean and a variance of 0.25. Note that source codes of the EGHR-*β* are appended as Supplementary Source Codes.

For Fig. 3: The performance of the EGHR-*β* is demonstrated using a natural image dataset. We prepare a total of 100 sources (*M* = 100): 12 high-variance colored-noise images with zero kurtosis, four low-variance natural images (hedgehogs), and 84 low-variance white-noise images with negative kurtosis. These sources consist of 200 × 200 pixels with RGB color. The natural images were retrieved from the Caltech101 dataset (http://www.vision.caltech.edu/Image_Datasets/Caltech101/)^40^, rescaled between 0 and 1, and adjusted to have a variance of 0.02. High-variance colored-noise images are created by enlarging 50 × 50 white noise images by a factor of four, and the original small-size images are produced by linearly summing truncated Gaussian noise (in the 0–1 range) and Laplace noise in order to have a mean of 0.5, variance of 0.023, and kurtosis of 0. Low-variance white-noise images are generated from a uniform distribution with a mean of 0.5 and variance of 0.002. We use these natural and noise images according to the protocol explained in^4,14^ by first subtracting the constant mean of 0.5 (i.e., the gray background). Each of 100 images (200 × 200 pixels, RGB) is treated as a vector (40, 000 pixels × 3 colors = 120, 000 dimensions). This source data composed of these 100 vectors (a 100 × 120, 000 matrix) is mixed by a 100 × 100-dimensional rotation matrix *R*. A column of the resulting input data is randomly sampled at each step for training (*T* = 3 × 10^7^ steps in total). The mixed signals are introduced as input into a one-layer feed-forward neural network (as in Fig. 1) to obtain the four-dimensional output (*N* = 4). Synaptic strength matrix *W* is updated by the EHGR-*β* with *β* = 0, 0.02, or 0.8. Hence, the input to output dimensions are

$${\rm{Input}}\,(100)\to {\rm{EGHR}}-\beta (4).$$

For a comparison, a two-layer feed-forward network is considered in which synaptic strengths in the first and second layers are updated by Oja’s subspace rule and Amari’s ICA rule, respectively. In this case, the input to intermediate representation to output dimensions are

$${\rm{Input}}\,(100)\to {\rm{Oja}}\mbox{'}{\rm{s}}\,{\rm{subspace}}\,{\rm{rule}}\,(4)\to {\rm{Amari}}\mbox{'}{\rm{s}}\,{\rm{ICA}}\,{\rm{rule}}(4).$$

The learning rate is defined by *η* = 2 × 10^−3^. For all algorithms, *R* is a common random rotation matrix, and *W* starts from the identity matrix.

## Electronic supplementary material

Supplementary Movie 1

Supplementary Information

scirep_fig2_eghr.c

scirep_functions.h

scirep_mathematics.h
