# Supplementary material for: Error-Gated Hebbian Rule: A Local Learning Rule for Principal and Independent Component Analysis
Source: Sci Rep. 2018 Jan 30;8:1835. doi: 10.1038/s41598-018-20082-0 (PMC5789861; doi:10.1038/s41598-018-20082-0)
Supplement: Supplementary file 2 — Supplementary Information [file 41598_2018_20082_MOESM2_ESM.pdf]

Supplementary Information

Error-Gated Hebbian Rule: A Local Learning Rule for Principal and Independent Component Analysis

Takuya Isomura, Taro Toyoizumi

Supplementary Figures

Figure S1
Figure S2

Supplementary Movie

Movie S1

Supplementary Source Codes

scirep_fig2_eghr.c
scirep_mathematics.h
scirep_functions.h

Supplementary Methods

S1. Lemma
S2. Fixed point of the EGHR-β
S3. Linear stability of the EGHR-β
S4. Conventional PCA algorithm

Supplementary Figures

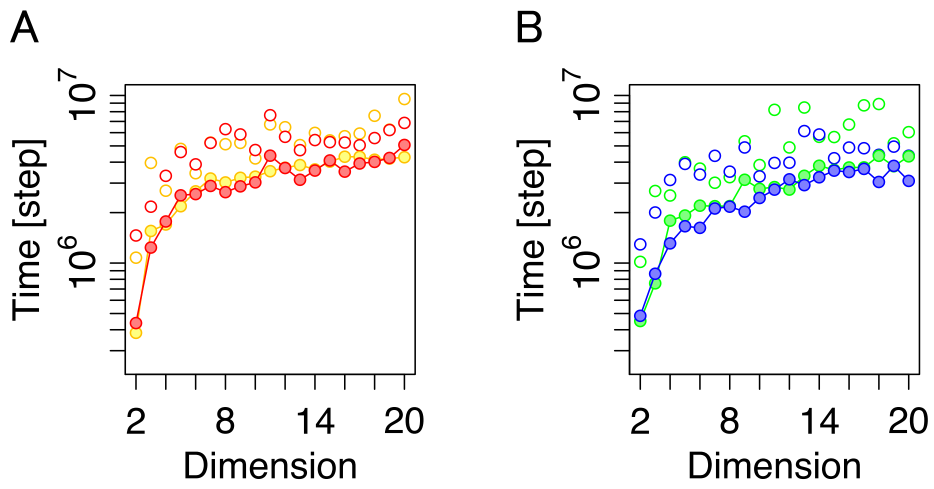

Figure S1. Dimension dependency of EGHR-β relaxation time with β = 0. The relaxation time is the time needed to perform ICA, where we define that ICA is achieved when the ratio of first to second maximum absolute values for every row and column of matrix $K = WA$ is less than threshold e_{th} = 0.1. The 2-to-20-dimensional sources follow a unit Laplace or uniform distribution, and mixing matrix A is a square matrix multiplying a distortion matrix and a random rotation matrix, where the distortion matrix is defined such that the covariance of inputs becomes cov(x_i, x_i) = 1 and cov(x_i, x_j) = 0.4 for $i \neq j$. The learning rate of $\eta = 1 \times 10^{-5}$ was used, and W was started from an identity matrix. Simulations were conducted 10 times for each dimension with different A. **(A)** Dimension dependency of the relaxation time with Laplace sources. Red filled and open circles are the median and maximum relaxation times, respectively. Yellow filled and open circles are the results of the original EHGR [4] for comparison. Note that the Laplace prior distribution $p_0(s_i) \propto \exp(-\sqrt{2}\,|s_i|)$ was assumed to calculate $g(\mathbf{u})$ and $E(\mathbf{u})$. **(B)** Dimension dependency of the relaxation time with uniform sources. Blue filled and open circles are the median and maximum relaxation times, respectively. Green filled and open circles are the results of the original EHGR [4]. Note that the prior distribution of $p_0(s_i) \propto \exp(-s_i^4/4)$ was assumed to calculate $g(\mathbf{u})$ and $E(\mathbf{u})$. Therefore, the EGHR-β as well as the original EGHR reliably perform ICA with random mixing matrices and the up-to-20-dimensional sources without being trapped in spurious solutions.

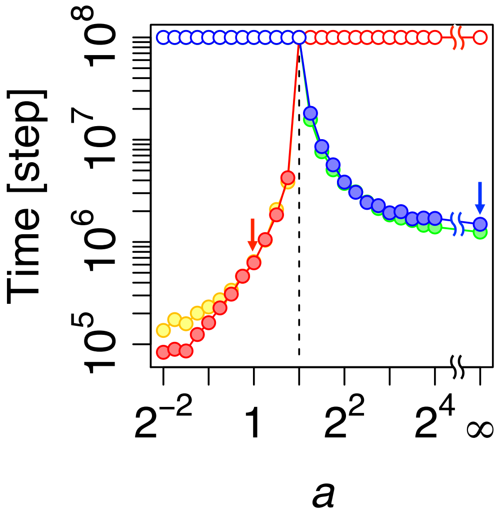

Figure S2. Robustness of EGHR-β with $\beta = 0$ to a choice of nonlinear function $g(\mathbf{u})$. Suppose, the two-dimensional sources follow $p(s_i) \propto \exp(-b\,|s_i|^a)$ ($a > 0$, b is defined such that the variance of s_i is one), and $A = (\cos\pi/6, -\sin\pi/6; \sin\pi/6, \cos\pi/6)$ is a rotation matrix. The same measure of relaxation time as Fig. S1 was plotted, but the threshold was defined as $e_{th} = 0.01$. The learning rate of $\eta = 1 \times 10^{-5}$ and the upper bound of simulation time $T = 1 \times 10^8$ were used, and W was started from an identity matrix. Red and blue circles represent relaxation times with non-linear functions $g(\mathbf{u})$ optimized for a Laplace distribution (thus $a = 1$; see the red arrow in the figure) and a uniform distribution ($a = \infty$; blue arrow), respectively. Filled circles indicate that ICA was successful with the non-linear function before the T-th step, while open circles indicate that ICA was not achieved before the T-th step. Yellow and green circles are the results of the original EGHR [4] with non-linear functions $g(\mathbf{u})$ optimized for a Laplace distribution and a uniform distribution, respectively. Therefore, the EGHR-β as well as the original EGHR robustly perform ICA for a range of a (except $a = 2$, where no ICA solution exists) by using $g(\mathbf{u})$ designed either for $a = 1$ or ∞.

Supplementary Movie

Supplementary Movie 1. Performance of EGHR-β with natural and noise images. See the Figure 3 legend and Methods for details.

Supplementary source codes

Supplementary Source Codes 1-3. C-language source codes of EGHR-β that demonstrate PCA and ICA. See the Figure 2 legend and Methods for details.

Supplementary Methods

S1. Lemma

S1.1 When $\langle \bullet \rangle = \int \bullet\, p_0(\mathbf{s})\, d\mathbf{s}$ and $g(s_i) = -\mathrm{d}\log p_0(s_i)/\mathrm{d}s_i$, $\langle \varphi(\mathbf{s}) g(\mathbf{s}) \rangle = \langle \varphi'(\mathbf{s}) \rangle$ holds for arbitrary function $\varphi(\mathbf{s})$.

[Proof]

Since $g(\mathbf{s}) = -p_0'(\mathbf{s}) / p_0(\mathbf{s})$, we have

$$\langle \varphi(\mathbf{s}) g(\mathbf{s}) \rangle = \int \varphi(\mathbf{s}) g(\mathbf{s})\, p_0(\mathbf{s})\, d\mathbf{s} = -\int \varphi(\mathbf{s})\, p_0'(\mathbf{s})\, d\mathbf{s} = \int \varphi'(\mathbf{s})\, p_0(\mathbf{s})\, d\mathbf{s} = \langle \varphi'(\mathbf{s}) \rangle.$$

[Proof end]

S1.2 Suppose an $M \times M$ square matrix C. When $s_1, \ldots, s_M$ independently follow even distributions $p(s_1), \ldots, p(s_M)$ with zero mean and unit variance, respectively, we have $\langle (\mathbf{s}^T C\, \mathbf{s})\, \mathbf{s}\mathbf{s}^T \rangle = C + C^T + \mathrm{tr}(C) I + \mathrm{Diag}[\kappa_i C_{ii}]$. Note that $\kappa_i \equiv \langle s_i^4 \rangle - 3$.

[Proof]

Suppose $G = \langle (\mathbf{s}^T C\, \mathbf{s})\, \mathbf{s}\mathbf{s}^T \rangle$. Since $\mathbf{s}^T C\, \mathbf{s} = \sum_k \sum_l C_{kl}\, s_k\, s_l$, an element of G is $G_{ij} = \langle \sum_k \sum_l C_{kl}\, s_k\, s_l\, s_i\, s_j \rangle$. When $i = j$, since $\langle s_i \rangle = \langle s_i^3 \rangle = 0$, $\langle s_i^2 \rangle = 1$, and $\langle s_i^4 \rangle = \kappa_i, + 3$, we have

$$G_{ii} = \langle \textstyle\sum_k C_{kk}\, s_k^2\, s_i^2 \rangle = \sum_{k \neq i} C_{kk} + C_{ii} \langle s_i^4 \rangle = 2C_{ii} + \kappa_i C_{ii} + \mathrm{tr}(C).$$

When $i \neq j$, we have

$$G_{ij} = \langle (C_{ij} + C_{ji})\, s_i^2\, s_j^2 \rangle = C_{ij} + C_{ji}.$$

Therefore, we get $G = C + C^T + \mathrm{tr}(C) I + \mathrm{Diag}[\kappa_i C_{ii}]$.

[Proof end]

S1.3 Suppose an $N \times M$ rectangle matrix C, an M-dimensional vector $\mathbf{s} = (s_1, \ldots, s_M)^T$, and an N-dimensional vector $\tilde{\mathbf{s}} = (s_1, \ldots, s_N)^T$. When $s_1, \ldots, s_N$ independently follow an identical even distribution $p_0(s_i)$ with zero mean and unit variance, $s_{N+1}, \ldots, s_M$ independently follow distributions with zero mean and unit variance, and $E(\tilde{\mathbf{s}}) - \langle E(\tilde{\mathbf{s}}) \rangle = -\log p_0(\tilde{\mathbf{s}}) + \langle \log p_0(\tilde{\mathbf{s}}) \rangle$ and $g'(s_i) = \partial^2 E(\tilde{\mathbf{s}})/\partial s_i^2$ hold, we have $\langle (E(\tilde{\mathbf{s}}) - \langle E(\tilde{\mathbf{s}}) \rangle) \mathrm{Diag}[g'(\tilde{\mathbf{s}})] C \mathbf{s}\mathbf{s}^T \rangle = \Omega \circ C$, where $\circ$ is Hadamard product, $\Omega_{ii} = \mathrm{cov}(-\log p_0(s_i), g'(s_i) s_i^2)$ for $i = j$, and $\Omega_{ij} = \mathrm{cov}(-\log p_0(s_i), g'(s_i)) + \mathrm{cov}(-\log p_0(s_j), s_j^2) \langle g'(s_i) \rangle \Theta[j \leq N]$ for $i \neq j$. Note that $\Theta[j \leq N]$ is 1 for $j \leq N$ and 0 otherwise.

[Proof]

Since $E(\tilde{\mathbf{s}})$ and $g'(s_i)$ are even functions,

$$\langle\, (E(\tilde{\mathbf{s}}) - \langle E(\tilde{\mathbf{s}})\rangle)\ \mathrm{Diag}[g'(\tilde{\mathbf{s}})]C\mathbf{s}\mathbf{s}^T \rangle_{ij} = \langle\, (E(\tilde{\mathbf{s}}) - \langle E(\tilde{\mathbf{s}})\rangle)\ g'(s_i) \sum_{l=1}^{M} C_{il} s_l s_j \,\rangle$$

$$= \langle\, (E(\tilde{\mathbf{s}}) - \langle E(\tilde{\mathbf{s}})\rangle)\ g'(s_i) s_j^2 \,\rangle C_{ij}.$$

We define $\Omega_{ij} = \langle\, (E(\tilde{\mathbf{s}}) - \langle E(\tilde{\mathbf{s}})\rangle)\ g'(s_i)s_j^2 \,\rangle$. When $i = j$,

$$\Omega_{ii} = \langle \sum_{k=1}^{N} (-\log p_0(s_k) + \langle \log p_0(s_k)\rangle) g'(s_i) s_i^2 \rangle = \langle (-\log p_0(s_i) + \langle \log p_0(s_i)\rangle) g'(s_i) s_i^2 \rangle$$

$$= \mathrm{cov}(-\log p_0(s_i),\ g'(s_i)s_i^2).$$

When $i \neq j$,

$$\Omega_{ij} = \langle \sum_{k=1}^{N} (-\log p_0(s_k) + \langle \log p_0(s_k)\rangle) g'(s_i) s_j^2 \rangle$$

$$= \langle \{-\log p_0(s_i) + \langle \log p_0(s_i)\rangle + (-\log p_0(s_j) + \langle \log p_0(s_j)\rangle)\Theta[j \le N]\} g'(s_i) s_j^2 \rangle$$

$$= \langle (-\log p_0(s_i) + \langle \log p_0(s_i)\rangle) g'(s_i) \rangle + \langle (-\log p_0(s_j) + \langle \log p_0(s_j)\rangle) s_j^2 \rangle \langle g'(s_i)\rangle \Theta[j \le N]$$

$$= \mathrm{cov}(-\log p_0(s_i),\ g'(s_i)) + \mathrm{cov}(-\log p_0(s_j),\ s_j^2) \langle g'(s_i)\rangle \Theta[j \le N].$$

[Proof end]

S2. Fixed point of the EGHR-β

Suppose that mixing matrix $A \in \mathbb{R}^{M\times M}$ consists of $A = R\ \Lambda^{1/2}\ B$ without loss of generality, where R and $B \in \mathbb{R}^{M\times M}$ are rotation matrices and $\Lambda \in \mathbb{R}^{M\times M}$ is a diagonal matrix. Moreover, suppose $s_1, \ldots, s_M$ independently follow even distributions $p(s_1), \ldots, p(s_M)$ with zero mean and unit variance. The cost function of the EGHR-β is given by

$$L \equiv \alpha \langle \frac{1}{2} (E(\mathbf{u}) - \langle E(\mathbf{u})\rangle)^2 - E(\mathbf{u}) \rangle + \frac{\beta}{2} \langle (E_u - E_x)^2 \rangle + \frac{\gamma}{2} \mathrm{tr}(W^T W), \qquad \text{(S1)}$$

where $E(\mathbf{u})$, E_x and E_u are defined by

$$E(\mathbf{u}) \equiv -\log p_0(\mathbf{u}),$$

$$E_u \equiv -\log N(\mathbf{u}) + \langle \log N(\mathbf{u})\rangle = \frac{1}{2} |\mathbf{u}|^2 - \frac{1}{2} \langle |\mathbf{u}|^2 \rangle = \frac{1}{2} \mathbf{u}^T\mathbf{u} - \frac{1}{2} \mathrm{tr}(K^T K),$$

$$E_x \equiv -\log N(\mathbf{x}) + \langle \log N(\mathbf{x}) \rangle = \frac{1}{2}|\mathbf{x}|^2 - \frac{1}{2}\langle |\mathbf{x}|^2 \rangle = \frac{1}{2}\mathbf{x}^T\mathbf{x} - \frac{1}{2}\mathrm{tr}(A^TA). \tag{S2}$$

Note that α, β, and γ are constants that control fixed points and the linear stability, in which $\alpha + \beta = 1$ holds without loss of generality. Partial derivative of L by W is given by

$$\frac{\partial L}{\partial W} = \alpha\langle (E(\mathbf{u}) - E_0)\, g(\mathbf{u})\, \mathbf{x}^T \rangle + \beta\langle (E_u - E_x)\, \mathbf{u}\, \mathbf{x}^T \rangle + \gamma W \tag{S3}$$

and the first-order differential form of L is given by

$$dL = \sum_{i=1}^{N}\sum_{j=1}^{M} \frac{\partial L}{\partial K_{ij}} dK_{ij}$$

$$= \sum_{i=1}^{N}\sum_{j=1}^{M} \{ \alpha\langle (E(\mathbf{u}) - E_0)\, g(u_i)\, s_j \rangle + \beta\langle (E_u - E_x)\, u_i\, s_j \rangle \}\, dK_{ij} + \gamma W_{ij}\, dW_{ij}$$

$$= \alpha\langle (E(\mathbf{u}) - E_0)\, g(\mathbf{u})^T d\mathbf{u} \rangle + \beta\langle (E_u - E_x)\, \mathbf{u}^T d\mathbf{u} \rangle + \gamma\mathrm{tr}(W^T dW). \tag{S4}$$

Note that $g(u_i) \equiv -\mathrm{d}\log p_0(u_i)/\mathrm{d}u_i$ and $E_0 \equiv \langle E(\mathbf{u}) \rangle + 1$. Since $\langle (\mathbf{s}^T C\, \mathbf{s})\, \mathbf{s}\mathbf{s}^T \rangle = C + C^T + \mathrm{tr}(C)I + \mathrm{Diag}[\kappa_i C_{ii}]$ (where $\kappa_i = \langle s_i^4 \rangle - 3$; see Supplementary Methods S1.2), we have

$$\langle (E_u - E_x)\, \mathbf{u}\, \mathbf{x}^T \rangle = \frac{1}{2}\langle (\, \mathbf{u}^T\mathbf{u} - \mathrm{tr}(K^TK) - \mathbf{x}^T\mathbf{x} + \mathrm{tr}(A^TA)\,)\, \mathbf{u}\, \mathbf{x}^T \rangle$$

$$= \frac{1}{2} K\langle (\, \mathbf{s}^T K^T K \mathbf{s} - \mathrm{tr}(K^TK) - \mathbf{s}^T A^T A \mathbf{s} + \mathrm{tr}(A^TA)\,)\, \mathbf{s}\, \mathbf{s}^T \rangle A^T$$

$$= K(\, K^TK + \frac{1}{2}\mathrm{Diag}[\kappa_i (K^TK)_{ii}] - A^TA - \frac{1}{2}\mathrm{Diag}[\kappa_i (A^TA)_{ii}]\,)A^T$$

$$= K(\, \Phi\circ(K^TK) - \Phi\circ(A^TA)\,)A^T, \tag{S5}$$

where $\circ$ is Hadamard product and $\Phi \in \mathbb{R}^{M\times M}$ is a symmetric matrix such that $\Phi_{ii} \equiv 1+\kappa_i/2$ for $i = j$ and $\Phi_{ij} \equiv 1$ for $i \neq j$. Thus, $\partial L/\partial W$ becomes

$$\frac{\partial L}{\partial W} = \alpha\langle (E(\mathbf{u}) - E_0)\, g(\mathbf{u})\, \mathbf{s}^T \rangle A^T + \beta K(\, \Phi\circ(K^TK) - \Phi\circ(A^TA)\,)A^T + \gamma K A^{-1} A^{-T} A^T. \tag{S6}$$

Obviously, $K = O$ is one of fixed points of Eq.(S6).

Case 1. Suppose $B = I$. When we use $E(\mathbf{u}) - \langle E(\mathbf{u}) \rangle = |\mathbf{u}|^2/2 - \langle |\mathbf{u}|^2 \rangle/2$, Eq.(S6) becomes

$$\frac{\partial L}{\partial W} = \alpha K(\, \Phi\circ(K^TK) - I\,)A^T + \beta K(\, \Phi\circ(K^TK) - \Phi\circ(A^TA)\,)A^T + \gamma K A^{-1} A^{-T} A^T$$

$$= K\{\, \Phi\circ(K^TK) - \beta\Phi\circ(A^TA) - \alpha I + \gamma (A^TA)^{-1}\, \}A^T$$

$$= K\{\ \Phi \circ (K^T K) - \beta \Phi \circ \Lambda - \alpha I + \gamma \Lambda^{-1}\ \} A^T. \tag{S7}$$

Since A is a regular matrix, from $\partial L/\partial W = O$, using a vector $\mathbf{1} = (1, \ldots, 1)^T \in \mathbb{R}^M$, a necessary and sufficient condition of a fixed point is given by

$$KK^T K = K\{(\mathbf{1}\mathbf{1}^T - \Phi) \circ (K^T K) + \beta \Phi \circ \Lambda + \alpha I - \gamma \Lambda^{-1}\}. \tag{S8}$$

We define a diagonal matrix $D \in \mathbb{R}^{M \times M}$ such that

$$D \equiv (\mathbf{1}\mathbf{1}^T - \Phi) \circ (K^T K) + \beta \Phi \circ \Lambda + \alpha I - \gamma \Lambda^{-1}$$

$$= \mathrm{Diag}[-\kappa_i/2\ (K^T K)_{ii} + \beta(1+\kappa_i/2)\ \Lambda_{ii} + \alpha - \gamma \Lambda_{ii}^{-1}] = \begin{pmatrix} D_1 & O \\ O & D_2 \end{pmatrix}, \tag{S9}$$

where diagonal matrices $D_1 \in \mathbb{R}^{N \times N}$ and $D_2 \in \mathbb{R}^{(M-N) \times (M-N)}$ are subsets of D. Moreover, $K \in \mathbb{R}^{N \times M}$ is represented as $K = P(I, Q)$ without loss of generality, where $P \in \mathbb{R}^{N \times N}$ is a regular matrix and $Q \in \mathbb{R}^{N \times (M-N)}$ is any matrix. Thus, Eq.(S8) becomes

$$P(I, Q) \begin{pmatrix} I \\ Q^T \end{pmatrix} P^T P(I, Q) = P(I, Q) \begin{pmatrix} D_1 & O \\ O & D_2 \end{pmatrix},$$

$$\Longleftrightarrow \quad (I + QQ^T) P^T P(I, Q) = (D_1, QD_2). \tag{S10}$$

Each block becomes $(I+QQ^T)\ P^T P = D_1$ and $(I+QQ^T)\ P^T P\ Q = Q\ D_2$. By substituting the former equation into the latter, we obtain $D_1\ Q = Q\ D_2$. Since this is one of Sylvester equations, it becomes $(D_1 \otimes I - I \otimes D_2)\mathrm{Vec}[Q] = O$, where $\otimes$ is the Kronecker product and $\mathrm{Vec}[Q] = (Q_{11}, \ldots, Q_{1(M-N)}, Q_{21}, \ldots, Q_{N(M-N)})^T$. If $(D_1 \otimes I - I \otimes D_2)$ is a regular matrix, $Q = O$ is a necessary and sufficient condition to satisfy the equation. Hereafter, suppose $Q = O$. Then, a necessary and sufficient condition of fixed point is

$$K = (P, O), \tag{S11}$$

where P is a regular matrix that holds

$$P^T P = D_1 = \mathrm{Diag}[-\kappa_i/2\ (P^T P)_{ii} + \beta(1+\kappa_i/2)\ \Lambda_{ii} + \alpha - \gamma \Lambda_{ii}^{-1}],$$

$$\Longleftrightarrow \quad \Phi_1 \circ (P^T P) = \beta \Phi_1 \circ \Lambda_1 + \alpha I - \gamma \Lambda_1^{-1},$$

$$\Longleftrightarrow \quad P^T P = \beta \Lambda_1 + \Phi_1^{\dagger} \circ (\alpha I - \gamma \Lambda_1^{-1}). \tag{S12}$$

Note that $\Phi_1 \in \mathbb{R}^{N \times N}$ is defined such that $(\Phi_1)_{ij} \equiv \Phi_{ij}$ for all $1 \le i, j \le N$ and $\Phi_1^{\dagger} \in \mathbb{R}^{N \times N}$

is defined such that $(\Phi_1^\dagger)_{ij} \equiv (\Phi_1)_{ij}^{-1}$ for all $1 \le i, j \le N$. When $(\alpha, \beta, \gamma) = (0, 1, 0)$, $P = J\Lambda_1^{1/2}$ with any rotation matrix $J \in \mathbb{R}^{N\times N}$ satisfies Eq.(S12). Thus, the EHGR obtain a subspace that N sources span in an equivalent way to the well-known Oja's subspace rule [11] except kurtosis term.

Case 2. Suppose $(\alpha, \beta, \gamma) = (1, 0, 0)$. Moreover, suppose $s_1, \ldots, s_N$ independently follow an identical distribution $p_0(s_i)$, and $s_{N+1}, \ldots, s_M$ are independent of each other. Under this condition, $K = (I, O) \in \mathbb{R}^{N\times M}$ is an equilibrium point of the EHGR-β, where I is the $N\times N$ identical matrix and O is the $N\times(M-N)$ zero matrix. This is confirmed by substituting $K = (I, O)$ into Eq.(S6). Using an N-dimensional vector $\tilde{\mathbf{s}} = (s_1, \ldots, s_N)^T$, $E(\mathbf{u})$ and E_0 become $E(\tilde{\mathbf{s}}) = -\log p_0(\tilde{\mathbf{s}})$ and $E_0 = \langle -\log p_0(\tilde{\mathbf{s}})\rangle + 1$, respectively. Since $\langle \varphi(\mathbf{s}) g(\tilde{\mathbf{s}})\rangle = \langle \varphi'(\mathbf{s})\rangle$ for arbitrary function $\varphi(\mathbf{s})$ from Supplementary Methods S1.1, we have

$$\frac{\partial L}{\partial W} = \langle (E(\tilde{\mathbf{s}}) - \langle E(\tilde{\mathbf{s}})\rangle - 1)\, g(\tilde{\mathbf{s}})\, \mathbf{s}^T\rangle A^T$$

$$= \langle g(\tilde{\mathbf{s}})\, \mathbf{s}^T + (E(\tilde{\mathbf{s}}) - \langle E(\tilde{\mathbf{s}})\rangle - 1)(I, O)\rangle A^T = O. \quad \text{(S13)}$$

Therefore, $K = (I, O)$ is a sufficient condition to be a fixed point of the EHGR-β. This fixed point of the EGHR-β is equivalent to that of the original EGHR [4] while M can be larger than N in this study.

Case 3. Suppose β and γ are small constants, $s_1, \ldots, s_N$ independently follow an identical even distribution $p_0(s_i)$ with zero mean and unit variance, and $s_{N+1}, \ldots, s_M$ independently follow distributions with zero mean and unit variance. Under this condition, as shown in the following, $K = (I, O) + \Delta = (I+\Delta_1, \Delta_2) \in \mathbb{R}^{N\times M}$ is a sufficient condition to be a fixed point of Eq.(S6), where I is the $N\times N$ identical matrix, O is the $N\times(M-N)$ zero matrix, $\Delta \equiv (\Delta_1, \Delta_2) \in \mathbb{R}^{N\times M}$ is a matrix with small absolute value elements, $|\Delta_{ij}| \ll 1$, and $\Delta_1 \in \mathbb{R}^{N\times N}$ and $\Delta_2 \in \mathbb{R}^{N\times(M-N)}$ are subsets of Δ. Using N-dimensional vector $\tilde{\mathbf{s}} = (s_1, \ldots, s_N)^T$, $g(\mathbf{u})$ becomes $g(\mathbf{u}) = g(\tilde{\mathbf{s}}+\Delta\mathbf{s}) \approx g(\tilde{\mathbf{s}}) + \mathrm{Diag}[g'(\tilde{\mathbf{s}})]\Delta\mathbf{s}$. Since $\langle (E(\tilde{\mathbf{s}}) - \langle E(\tilde{\mathbf{s}})\rangle - 1)\, g(\tilde{\mathbf{s}})\, \mathbf{s}^T\rangle = O$ and $\langle \varphi(\mathbf{s}) g(\tilde{\mathbf{s}})\rangle = \langle \varphi'(\mathbf{s})\rangle$ for arbitrary function $\varphi(\mathbf{s})$, the first term in the right side of Eq.(S6) becomes

$$\langle (E(\mathbf{u})-E_0) g(\mathbf{u})\mathbf{s}^T\rangle \approx \langle \{E(\tilde{\mathbf{s}}) - \langle E(\tilde{\mathbf{s}})\rangle + g(\tilde{\mathbf{s}})^T\Delta\mathbf{s} - \langle g(\tilde{\mathbf{s}})^T\Delta\mathbf{s}\rangle - 1\}(g(\tilde{\mathbf{s}}) + \mathrm{Diag}[g'(\tilde{\mathbf{s}})]\Delta\mathbf{s})\mathbf{s}^T\rangle$$

$$\approx \langle (g(\tilde{\mathbf{s}})^T\Delta\mathbf{s} - \langle g(\tilde{\mathbf{s}})^T\Delta\mathbf{s}\rangle)\, g(\tilde{\mathbf{s}})\, \mathbf{s}^T\rangle + \langle (E(\tilde{\mathbf{s}}) - \langle E(\tilde{\mathbf{s}})\rangle - 1)\, \mathrm{Diag}[g'(\tilde{\mathbf{s}})]\Delta\mathbf{s}\, \mathbf{s}^T\rangle$$
$$= \langle (\mathrm{Diag}[g'(\tilde{\mathbf{s}})]\Delta\mathbf{s} + (I,O)\Delta^T g(\tilde{\mathbf{s}}))\, \mathbf{s}^T\rangle + \langle g(\tilde{\mathbf{s}})^T\Delta\mathbf{s} - \langle g(\tilde{\mathbf{s}})^T\Delta\mathbf{s}\rangle\rangle (I,O)$$
$$+ \langle (E(\tilde{\mathbf{s}}) - \langle E(\tilde{\mathbf{s}})\rangle - 1)\, \mathrm{Diag}[g'(\tilde{\mathbf{s}})]\Delta\mathbf{s}\, \mathbf{s}^T\rangle$$
$$= \langle (I,O)\Delta^T g(\tilde{\mathbf{s}})\, \mathbf{s}^T\rangle + \langle (E(\tilde{\mathbf{s}}) - \langle E(\tilde{\mathbf{s}})\rangle)\, \mathrm{Diag}[g'(\tilde{\mathbf{s}})]\Delta\mathbf{s}\, \mathbf{s}^T\rangle$$
$$= (I,O)\Delta^T(I,O) + \Omega\circ\Delta$$
$$= (\Delta_1^T + \Omega_1\circ\Delta_1,\ \Omega_2\circ\Delta_2). \qquad \text{(S14)}$$

Note that from Supplementary Methods S1.3, $\Omega \equiv (\Omega_1, \Omega_2) \in \mathbb{R}^{N\times M}$ is defined such that $\Omega_{ii} \equiv \mathrm{cov}(-\log p_0(s_i), g'(s_i)s_i^2)$ for $i = j$ and $\Omega_{ij} \equiv \mathrm{cov}(-\log p_0(s_i), g'(s_i)) + \mathrm{cov}(-\log p_0(s_j), s_j^2)\langle g'(s_i)\rangle\Theta[j\le N]$ for $i \ne j$ ($\Theta[j\le N]$ is 1 for $j \le N$ and 0 otherwise). Note that $\Omega_1 \in \mathbb{R}^{N\times N}$ and $\Omega_2 \in \mathbb{R}^{N\times(M-N)}$ are subsets of Ω. We define $H \equiv (H_1, H_2; H_3, H_4) = A^TA \in \mathbb{R}^{M\times M}$. Since Δ, β, and γ are small, the second and third terms in the right side of Eq.(S6) is approximated as $\beta(I,O)(\ \Phi\circ((I,O)^T(I,O)) - \Phi\circ(A^TA)\ )A^T + \gamma(I,O)(A^TA)^{-1}A^T = (I,O)(\beta\Phi\circ(I-H) + \gamma H^{-1})A^T$. Therefore, Eq.(S6) becomes

$$\frac{\partial L}{\partial W} \approx (\Delta_1^T + \Omega_1\circ\Delta_1,\ \Omega_2\circ\Delta_2)A^T - (I,O)(\beta\Phi\circ(H-I) - \gamma H^{-1})A^T$$
$$= [(\Delta_1^T + \Omega_1\circ\Delta_1,\ \Omega_2\circ\Delta_2)$$
$$- (I,O)\begin{pmatrix} \beta\Phi_1\circ(H_1 - I) - \gamma(H^{-1})_1 & \beta H_2 - \gamma(H^{-1})_2 \\ \beta H_3 - \gamma(H^{-1})_3 & \beta\Phi_2\circ(H_4 - I) - \gamma(H^{-1})_4 \end{pmatrix}]\, A^T$$
$$= [(\Delta_1^T + \Omega_1\circ\Delta_1,\ \Omega_2\circ\Delta_2) - (\beta\Phi_1\circ(H_1 - I) - \gamma(H^{-1})_1,\ \beta H_2 - \gamma(H^{-1})_2)]\, A^T. \qquad \text{(S15)}$$

To be an equilibrium point, Δ needs to satisfy

$$\Delta_1^T + \Omega_1\circ\Delta_1 = \beta\Phi_1\circ(H_1 - I) - \gamma(H^{-1})_1 \quad \text{and} \quad \Omega_2\circ\Delta_2 = \beta H_2 - \gamma(H^{-1})_2. \qquad \text{(S16)}$$

Since the right side of the first equation is a symmetric matrix, if and only if Δ_1 is a symmetric matrix, a solution exists. Suppose Δ_1 is a symmetric matrix. Therefore, using a vector $\mathbf{1} = (1, \ldots, 1)^T \in \mathbb{R}^N$, we obtain

$$\Delta_1 = (\mathbf{1}\mathbf{1}^T + \Omega_1)^\dagger \circ \{\beta\Phi_1\circ(H_1 - I) - \gamma(H^{-1})_1\},$$
$$\Delta_2 = \Omega_2^\dagger \circ \{\beta H_2 - \gamma(H^{-1})_2\}. \qquad \text{(S17)}$$

Therefore, $K = (I+\Delta_1, \Delta_2)$ is a sufficient condition of a fixed point. Since $|\Delta_{ij}| \ll 1$, this fixed point is sufficiently close to $\tilde{\mathbf{s}} = (s_1, \ldots, s_N)^T$, thus, the EGHR-β can perform PCA and ICA simultaneously. Specifically, if $B = I$, Δ becomes a diagonal matrix and

its (i, i) element is $\Delta_{ii} = (1+\Omega_{ii})^{-1}$ $\{\beta\ \Phi_{ii}\ (\Lambda_{ii}-1) - \gamma\ \Lambda_{ii}^{-1}\}$; thus, an exact solution of PCA and ICA exists.

To see how the shape of the source distribution affects on the fixed point, suppose $p_0(s_i)$ ($1 \le i \le N$) follow $p_0(s_i) = \exp(-b|s_i|^a)/Z$, where $a > 0$ is a positive constant, $b > 0$ is tuned in a manner to hold $\langle s_i^2 \rangle = 1$, and Z is a partition function such that $Z = \int \exp(-b|s_i|^a) ds_i$. Note that s_{N+1}, …, s_M follow distributions with zero mean and unit variance. Since $-\log p_0(s_i) = b|s_i|^a$ + const., $g(s_i) = -\mathrm{d}\log p_0(s_i)/\mathrm{d}s_i = ba|s_i|^{a-1}\mathrm{sgn}(s_i)$, and $g'(s_i) = -\mathrm{d}g(s_i)/\mathrm{d}s_i = ba(a-1)|s_i|^{a-2} + ba|s_i|^{a-1}\delta(s_i)$, we have $-\log p_0(s_i) = g(s_i)s_i/a$ + const., $g'(s_i)s_i = (a-1)g(s_i)$ and $-\log p_0(s_i)\ g'(s_i) = (a-1)/a\ g(s_i)^2$ + const. $g'(s_i)$. Thus, Ω_{ij} becomes

For $i = j$,

$$\Omega_{ii} = \mathrm{cov}(-\log p_0(s_i), g'(s_i)s_i^2) = \langle(-\log p_0(s_i)+\langle\log p_0(s_i)\rangle)\ g'(s_i)s_i^2\rangle$$
$$= \langle(g(s_i)s_i/a - \langle g(s_i)s_i/a\rangle)\ (a-1)g(s_i)s_i\rangle$$
$$= (a-1)/a\ \langle(g'(s_i)s_i+g(s_i))\ s_i\rangle + (a-1)/a\ \langle g(s_i)s_i - \langle g(s_i)s_i\rangle\rangle$$
$$= (a-1)/a\ \langle(a-1)g(s_i)s_i + g(s_i)s_i\rangle = a-1,$$

For $i \ne j \le N$,

$$\Omega_{ij} = \mathrm{cov}(-\log p_0(s_i), g'(s_i)) + \mathrm{cov}(-\log p_0(s_j), s_j^2)\langle g'(s_i)\rangle$$
$$= \langle(-\log p_0(s_i)+\langle\log p_0(s_i)\rangle)\ g'(s_i)\rangle + \langle(-\log p_0(s_j)+\langle\log p_0(s_j)\rangle)\ s_j^2\rangle\ \langle g'(s_i)\rangle$$
$$= \langle(a-1)/a\ g(s_i)^2 - \langle g(s_i)s_i/a\rangle\ g'(s_i)\rangle + \langle(g(s_j)s_j/a - \langle g(s_j)s_j/a\rangle)\ s_j^2\rangle\ \langle g'(s_i)\rangle$$
$$= (a-2)/a\ \langle g'(s_i)\rangle + 2/a\ \langle g'(s_i)\rangle = \langle g'(s_i)\rangle,$$

For $N+1 \le j \le M$,

$$\Omega_{ij} = \mathrm{cov}(-\log p_0(s_i), g'(s_i)) = (a-2)/a\ \langle g'(s_i)\rangle. \qquad \text{(S18)}$$

S3. Linear stability of the EGHR-β

The second derivative of L is given by

$$
\begin{aligned}
d^2L &= \sum_{k=1}^{N}\sum_{l=1}^{M} \frac{\partial dL}{\partial K_{kl}} dK_{kl} \\
&= \alpha \langle (g(\mathbf{u})^T d\mathbf{u} - \langle g(\mathbf{u})^T d\mathbf{u}\rangle)\, g(\mathbf{u})^T d\mathbf{u} + (E(\mathbf{u}) - E_0)\, d\mathbf{u}^T \mathrm{Diag}[g'(\mathbf{u})]\, d\mathbf{u} \rangle \\
&\quad + \beta \langle (\mathbf{u}^T d\mathbf{u} - \langle \mathbf{u}^T d\mathbf{u}\rangle)\, \mathbf{u}^T d\mathbf{u} + (E_u - E_x)\, d\mathbf{u}^T d\mathbf{u} \rangle + \gamma\, \mathrm{tr}(dW^T dW) \\
&= \alpha \langle (\mathbf{s}^T dK^T g(\mathbf{u}) - \langle \mathbf{s}^T dK^T g(\mathbf{u})\rangle)\, \mathbf{s}^T dK^T g(\mathbf{u}) + (E(\mathbf{u}) - E_0)\, \mathbf{s}^T dK^T \mathrm{Diag}[g'(\mathbf{u})]\, dK\, \mathbf{s}\rangle \\
&\quad + \beta \langle (\mathbf{s}^T dK^T K\mathbf{s} - \langle \mathbf{s}^T dK^T K\mathbf{s}\rangle)\, \mathbf{s}^T dK^T K\mathbf{s} + (E_u - E_x)\, \mathbf{s}^T dK^T dK\mathbf{s}\rangle + \gamma\, \mathrm{tr}(A^{-T} dK^T dK A^{-1}) \\
&= \alpha\, \mathrm{tr}[\, \langle (\mathbf{s}^T dK^T g(\mathbf{u}) - \langle \mathbf{s}^T dK^T g(\mathbf{u})\rangle) g(\mathbf{u})\mathbf{s}^T + (E(\mathbf{u}) - E_0)\, \mathrm{Diag}[g'(\mathbf{u})]\, dK\, \mathbf{s}\mathbf{s}^T\rangle dK^T\,] \\
&\quad + \beta\, \mathrm{tr}[\, \langle (\mathbf{s}^T dK^T K\, \mathbf{s} - \langle \mathbf{s}^T dK^T K\, \mathbf{s}\rangle)\mathbf{s}\mathbf{s}^T\rangle dK^T K + \langle (E_u - E_x)\mathbf{s}\mathbf{s}^T\rangle dK^T dK\,] \\
&\quad + \gamma\, \mathrm{tr}[A^{-1} A^{-T} dK^T dK]. \qquad \text{(S19)}
\end{aligned}
$$

From Supplementary Methods S1.2, $\langle (\mathbf{s}^T dK^T K\, \mathbf{s} - \langle \mathbf{s}^T dK^T K\, \mathbf{s}\rangle)\mathbf{s}\mathbf{s}^T \rangle = dK^T K + K^T dK + \mathrm{Diag}[\kappa_i (dK^T K)_{ii}] = \Phi\circ(dK^T K + K^T dK)$ (where $\Phi_{ii} = 1+\kappa_i/2$ for $i = j$ and $\Phi_{ij} = 1$ for $i \neq j$). Moreover, by Eq.(S5), $\langle (E_u - E_x)\, \mathbf{s}\mathbf{s}^T\rangle = K^T K + \mathrm{Diag}[\kappa_i (K^T K)_{ii}]/2 - A^T A - \mathrm{Diag}[\kappa_i (A^T A)_{ii}]/2 = \Phi\circ(K^T K - A^T A)$. Thus, the second term of Eq.(S19) becomes

$$
\begin{aligned}
&\mathrm{tr}[\, \langle (\mathbf{s}^T dK^T K\, \mathbf{s} - \langle \mathbf{s}^T dK^T K\, \mathbf{s}\rangle)\mathbf{s}\mathbf{s}^T\rangle dK^T K + \langle (E_u - E_x)\mathbf{s}\mathbf{s}^T\rangle dK^T dK\,] \\
&\quad = \mathrm{tr}[\, \{\Phi\circ(dK^T K + K^T dK)\}\, dK^T K + \{\Phi\circ(K^T K - A^T A)\}\, dK^T dK\,]. \qquad \text{(S20)}
\end{aligned}
$$

Therefore, d^2L becomes

$$
\begin{aligned}
d^2L &= \alpha\, \mathrm{tr}[\, \langle (\mathbf{s}^T dK^T g(\mathbf{u}) - \langle \mathbf{s}^T dK^T g(\mathbf{u})\rangle) g(\mathbf{u})\mathbf{s}^T + (E(\mathbf{u}) - E_0)\, \mathrm{Diag}[g'(\mathbf{u})]\, dK\, \mathbf{s}\mathbf{s}^T\rangle dK^T\,] \\
&\quad + \beta\, \mathrm{tr}[\{\Phi\circ(dK^T K + K^T dK)\}\, dK^T K] + \mathrm{tr}[\{\beta\Phi\circ(K^T K - A^T A) + \gamma A^{-1} A^{-T}\}\, dK^T dK]. \qquad \text{(S21)}
\end{aligned}
$$

Case 1. Suppose $B = I$. When we use $E(\mathbf{u}) - \langle E(\mathbf{u})\rangle = |\mathbf{u}|^2/2 - \langle |\mathbf{u}|^2\rangle/2$, $K = (P, O)$ gives an equilibrium point of the cost function wherever P satisfies Eq.(S12). The first term of Eq.(S21) becomes

$$
\begin{aligned}
&\mathrm{tr}[\langle (\mathbf{s}^T dK^T g(\mathbf{u}) - \langle \mathbf{s}^T dK^T g(\mathbf{u})\rangle) g(\mathbf{u})\mathbf{s}^T + (E(\mathbf{u}) - E_0)\, \mathrm{Diag}[g'(\mathbf{u})]\, dK\, \mathbf{s}\mathbf{s}^T\rangle dK^T] \\
&\quad = \mathrm{tr}[\langle (\mathbf{s}^T dK^T \mathbf{u} - \langle \mathbf{s}^T dK^T \mathbf{u}\rangle)\mathbf{u}\mathbf{s}^T + (\mathbf{u}^T\mathbf{u}/2 - \mathrm{tr}(K^T K)/2 - 1)\, dK\, \mathbf{s}\mathbf{s}^T\rangle dK^T] \\
&\quad = \mathrm{tr}[K\langle (\mathbf{s}^T dK^T K\, \mathbf{s} - \mathrm{tr}(dK^T K))\mathbf{s}\mathbf{s}^T\rangle dK^T + dK\langle (\mathbf{s}^T K^T K\mathbf{s}/2 - \mathrm{tr}(K^T K)/2 - 1)\mathbf{s}\mathbf{s}^T\rangle dK^T] \\
&\quad = \mathrm{tr}[K(dK^T K + K^T dK + \mathrm{Diag}[\kappa_i (dK^T K)_{ii}])dK^T + dK(K^T K + \mathrm{Diag}[\kappa_i (K^T K)_{ii}]/2 - I)dK^T] \\
&\quad = \mathrm{tr}[\, \Phi\circ(dK^T K + K^T dK)\, dK^T K + (\Phi\circ(K^T K) - I)\, dK^T dK\,]. \qquad \text{(S22)}
\end{aligned}
$$

Thus, by supposing $K = (P, O)$ and $dK = (dK_1, dK_2)$, d^2L becomes

$$
\begin{aligned}
d^2L &= \mathrm{tr}[(\Phi\circ(dK^TK))dK^TK] + \mathrm{tr}[(\Phi\circ(K^TdK))dK^TK] \\
&\quad + \mathrm{tr}[(\Phi\circ(K^TK - \beta A^TA) - \alpha I + \gamma A^{-1}A^{-T})dK^TdK] \\
&= \mathrm{tr}[(\Phi_1\circ(dK_1{}^TP))dK_1{}^TP] + \mathrm{tr}[(\Phi_1\circ(P^TdK_1),P^TdK_2)(P^TdK_1,P^TdK_2)^T] \\
&\quad + \mathrm{tr}[\begin{pmatrix} \Phi_1\circ(P^TP-\beta\Lambda_1)-\alpha I+\gamma\Lambda_1^{-1} & O \\ O & -\beta\Phi_2\circ\Lambda_2-\alpha I+\gamma\Lambda_2^{-1} \end{pmatrix} dK^TdK] \\
&= \mathrm{tr}[(\Phi_1\circ(dK_1{}^TP + P^TdK_1))dK_1{}^TP] + \mathrm{tr}[(\Phi_1\circ(P^TP-\beta\Lambda_1) - \alpha I + \gamma\Lambda_1{}^{-1})dK_1{}^TdK_1] \\
&\quad + \mathrm{tr}[P^TdK_2dK_2{}^TP] + \mathrm{tr}[(-\beta\Phi_2\circ\Lambda_2 - \alpha I + \gamma\Lambda_2{}^{-1})dK_2{}^TdK_2]. \qquad \text{(S23)}
\end{aligned}
$$

Since $\Phi_1\circ(P^TP) = \beta\Phi_1\circ\Lambda_1 + \alpha I - \gamma\Lambda_1{}^{-1}$ (see Eq.(S12)) and $\Phi_1\circ(dK_1{}^TP + P^TdK_1) = dK_1{}^TP + P^TdK_1 + \mathrm{Diag}[\kappa_i(dK_1{}^TP)_{ii}]$, d^2L becomes

$$
\begin{aligned}
d^2L &= \mathrm{tr}[(dK_1{}^TP + P^TdK_1)dK_1{}^TP] + \sum_{i=1}^{N} \kappa_i(dK_1{}^TP)_{ii}{}^2 \\
&\quad + \mathrm{tr}[P^TdK_2dK_2{}^TP] + \mathrm{tr}[(-\beta\Phi_2\circ\Lambda_2 - \alpha I + \gamma\Lambda_2{}^{-1})dK_2{}^TdK_2] \\
&= \mathrm{tr}[(dX_1{}^T + dX_1)dX_1{}^T] + \sum_{i=1}^{N} \kappa_i(dX_1{}^T)_{ii}{}^2 \\
&\quad + \mathrm{tr}[dX_2dX_2{}^T] + \mathrm{tr}[(-\beta\Phi_2\circ\Lambda_2 - \alpha I + \gamma\Lambda_2{}^{-1})dX_2{}^T(P^{-1}P^{-T})dX_2] \\
&= \frac{1}{2}\sum_{i=1}^{N}\sum_{j\neq i\leq N} (dX_{ij} + dX_{ji})^2 + 2\sum_{i=1}^{N} \Phi_{ii}(dX_1)_{ii}{}^2 \\
&\quad + \mathrm{tr}[dX_2dX_2{}^T] - \mathrm{tr}[(P^TP)^{-1}dX_2(\beta\Phi_2\circ\Lambda_2 - \alpha I + \gamma\Lambda_2{}^{-1})dX_2{}^T]. \qquad \text{(S24)}
\end{aligned}
$$

Note that $dX = (dX_1, dX_2) = P^TdK = P^T(dK_1, dK_2)$ is supposed. Because coefficients of dX_1 are positive, the linear stability depends on the sign of coefficients of dX_2. Since $P^TP = \beta\Lambda_1 + \Phi_1{}^\dagger\circ(\alpha I - \gamma\Lambda_1{}^{-1})$ and $(\beta\Phi_2\circ\Lambda_2 - \alpha I + \gamma\Lambda_2{}^{-1})$ are diagonal matrices, we have

$$
\begin{aligned}
&\mathrm{tr}[dX_2dX_2{}^T] - \mathrm{tr}[(P^TP)^{-1}dX_2(\beta\Phi_2\circ\Lambda_2 - \alpha I + \gamma\Lambda_2{}^{-1})dX_2{}^T] \\
&= \sum_{i=1}^{N}\sum_{j=N+1}^{M} dX_{ij}{}^2 - \sum_{i=1}^{N}\sum_{j=N+1}^{M} ((P^TP)^{-1})_{ii}\, dX_{ij}{}^2\, (\beta\Phi_2\circ\Lambda_2 - \alpha I + \gamma\Lambda_2{}^{-1})_{jj} \\
&= \sum_{i=1}^{N}\sum_{j=N+1}^{M} ((P^TP)^{-1})_{ii}\, dX_{ij}{}^2\, \{ (\beta\Lambda_1+\Phi_1{}^\dagger\circ(\alpha I-\gamma\Lambda_1{}^{-1}))_{ii} - (\beta\Phi_2\circ\Lambda_2-\alpha I+\gamma\Lambda_2{}^{-1})_{jj} \}
\end{aligned}
$$

$$= \sum_{i=1}^{N}\sum_{j=N+1}^{M} ((P^TP)^{-1})_{ii}\, dX_{ij}^2 \;\{\; \beta\Lambda_{ii} + \Phi_{ii}^{-1}(\alpha-\gamma\Lambda_{ii}^{-1}) - (\beta\Phi_{jj}\Lambda_{jj} - \alpha + \gamma\Lambda_{jj}^{-1}) \;\}$$

$$= \sum_{i=1}^{N}\sum_{j=N+1}^{M} ((P^TP)^{-1})_{ii}\, dX_{ij}^2 \;\{\; \beta(\Lambda_{ii}-\Phi_{jj}\Lambda_{jj}) + \alpha(\Phi_{ii}^{-1}-1) - \gamma(\Phi_{ii}^{-1}\Lambda_{ii}^{-1}-\Lambda_{jj}^{-1}) \;\} \quad \text{(S25)}$$

Therefore, a necessary and sufficient condition for linear stability is

$$\beta(\Lambda_{ii}-\Phi_{jj}\Lambda_{jj}) + \alpha(\Phi_{ii}^{-1}-1) - \gamma(\Phi_{ii}^{-1}\Lambda_{ii}^{-1}-\Lambda_{jj}^{-1}) > 0 \quad \text{for } 1\le i\le N,\ N+1\le j\le M. \quad \text{(S26)}$$

When $(\alpha, \beta, \gamma) = (0, 1, 0)$ and $s_{N+1}, \ldots, s_M$ follow a unit Gaussian distribution, the condition for linear stability is $\Lambda_{ii} > \Lambda_{jj}$ for $1 \le i \le N$ and $N+1 \le j \le M$. Thus, when **u** represents a space that the first to Nth components span, the state is stable, while when **u** involves other components, the state is unstable. Accordingly, the EGHR-β can extract principal components. However, when $s_{N+1}, \ldots, s_M$ follow non-Gaussian distributions, the linear stability condition depends on kurtosis of the distributions.

Case 2. Suppose $(\alpha, \beta, \gamma) = (1, 0, 0)$, $s_1, \ldots, s_N$ independently follow an even identical distribution $p_0(s_i)$ with zero mean and unit variance, and $s_{N+1}, \ldots, s_M$ independently follow distributions with zero mean and unit variance. Under this condition, $K = (I, O)$ is an equilibrium point. When $K = (I, O)$, d^2L becomes

$$d^2L = \mathrm{tr}[\langle(\mathbf{s}^T dK^T g(\tilde{\mathbf{s}}) - \langle \mathbf{s}^T dK^T g(\tilde{\mathbf{s}})\rangle) g(\tilde{\mathbf{s}})\mathbf{s}^T + (E(\tilde{\mathbf{s}}) - \langle E(\tilde{\mathbf{s}})\rangle - 1)\,\mathrm{Diag}[g'(\tilde{\mathbf{s}})]\, dK\, \mathbf{s}\mathbf{s}^T\rangle dK^T]$$
$$= \mathrm{tr}[\langle((I,O)dK^T g(\tilde{\mathbf{s}}) + \mathrm{Diag}[g'(\tilde{\mathbf{s}})]dK\mathbf{s})\mathbf{s}^T + (E(\tilde{\mathbf{s}}) - \langle E(\tilde{\mathbf{s}})\rangle - 1)\,\mathrm{Diag}[g'(\tilde{\mathbf{s}})]\, dK\, \mathbf{s}\mathbf{s}^T\rangle dK^T]$$
$$= \mathrm{tr}[\; (I,O)dK^T(I,O)dK^T + \langle(E(\tilde{\mathbf{s}}) - \langle E(\tilde{\mathbf{s}})\rangle)\,\mathrm{Diag}[g'(\tilde{\mathbf{s}})]\, dK\, \mathbf{s}\mathbf{s}^T\rangle dK^T \;]$$
$$= \mathrm{tr}[\; dK_1 dK_1 + (\Omega \circ dK)\, dK^T \;]$$
$$= \sum_{i=1}^{N}(1+\Omega_{ii})dK_{ii}^2 + \sum_{i=1}^{N}\sum_{i\ne j\le N}(dK_{ij}dK_{ji} + \Omega_{ij}dK_{ij}^2) + \sum_{i=1}^{N}\sum_{j=N+1}^{M}\Omega_{ij}\, dK_{ij}^2$$
$$= \sum_{i=1}^{N}(1+\Omega_{ii})dK_{ii}^2 + \frac{1}{2}\sum_{i=1}^{N}\sum_{i\ne j\le N}(\Omega_{ij}dK_{ij}^2 + 2dK_{ij}dK_{ji} + \Omega_{ji}dK_{ji}^2) + \sum_{i=1}^{N}\sum_{j=N+1}^{M}\Omega_{ij}\, dK_{ij}^2. \quad \text{(S27)}$$

Therefore, necessary and sufficient conditions to be linearly stable are given by

$$1 + \Omega_{ii} > 0 \quad \text{for } 1 \le i = j \le N,$$
$$\Omega_{ij}\,\Omega_{ji} > 1 \quad \text{for } 1 \le i \ne j \le N,$$
$$\Omega_{ij} > 0 \quad \text{for } 1 \le i \le N,\ N+1 \le j \le M. \quad \text{(S28)}$$

To see how the shape of the source distribution affects on the linear stability, again

suppose $p_0(s_i)$ $(1 \le i \le N)$ to follow $p_0(s_i) = \exp(-b|s_i|^a)/Z$ and solve Ω_{ij} as shown in Eq.(S18). Then, whenever $a \ge 1$, $1 + \Omega_{ii} = a > 0$ and $\Omega_{ij}\,\Omega_{ji} = \langle g'(s_i)\rangle^2 > 1$ hold. Whereas, $\Omega_{ij} = (a-2)/a\ \langle g'(s_i)\rangle > 0$ holds only when $a > 2$. Therefore, $a > 2$ is a necessary and sufficient condition to be linearly stable. Accordingly, when $s_1, \ldots, s_N$ follow a sub-Gaussian distribution ($a > 2$) and $s_{N+1}, \ldots, s_M$ follow Gaussian or super-Gaussian distributions, $s_1, \ldots, s_N$ are chosen as outputs. Whereas, when $s_1, \ldots, s_N$ follow a super-Gaussian distribution ($a < 2$) and $s_{N+1}, \ldots, s_M$ follow Gaussian or sub-Gaussian distributions, some of $s_1, \ldots, s_N$ may not be chosen while some of $s_{N+1}, \ldots, s_M$ may be chosen as outputs.

Case 3. Suppose that mixing matrix $A \in \mathbb{R}^{N\times N}$ consists of $A = R\,\Lambda^{1/2}$, where $R \in \mathbb{R}^{N\times N}$ is a rotation matrix and $\Lambda \in \mathbb{R}^{N\times N}$ is a diagonal matrix. Then, H becomes $H = A^TA \approx \Lambda$. Moreover, suppose $|\beta|, |\gamma| << 1$ and $\alpha = 1 - \beta \approx 1$. From the fixed point analysis, when $B = I$, a fixed point is represented as $\mathbf{u} = (I+\Delta_1,\ O)\ \mathbf{s}$, where $\Delta_1 \in \mathbb{R}^{N\times N}$ is a diagonal matrix defined by $\Delta_1 = (\mathbf{1}\mathbf{1}^T+\Omega_1)^{\dagger}\circ\{\beta\Phi_1\circ(\Lambda_1 - I) - \gamma\Lambda_1^{-1}\}$, while $\Delta_2 = O$. The first term of Eq.(S21) becomes

$$\begin{aligned}
&\langle(\mathbf{s}^T dK^T g(\mathbf{u}) - \langle\mathbf{s}^T dK^T g(\mathbf{u})\rangle)g(\mathbf{u})\mathbf{s}^T + (E(\mathbf{u})-E_0)\ \mathrm{Diag}[g'(\mathbf{u})]\ dK\ \mathbf{s}\mathbf{s}^T\rangle_{ij} \\
&\quad = \langle(\textstyle\sum_{k,l} g(u_k)\ dK_{kl}\ s_l - \langle\sum_{k,l} g(u_k)\ dK_{kl}\ s_l\rangle)\ g(u_i)\ s_j + (E(\mathbf{u})-E_0)\ g'(u_i) \sum_l dK_{il}\ s_l\ s_j\rangle \\
&\quad = \langle(\textstyle\sum_{k,l} g(u_k)\ dK_{kl}\ s_l - \langle\sum_{k,l} g(u_k)\ dK_{kl}\ s_l\rangle)\ g(u_i)\ s_j + (E(\mathbf{u})-E_0)\ g'(u_i)\ dK_{ij}\ s_j^2\rangle. \qquad \text{(S29)}
\end{aligned}$$

To further calculate Eq.(S29), again suppose $p_0(s_i)$ $(1 \le i \le N)$ follow $p_0(s_i) = \exp(-b|s_i|^a)/Z$. Thus, $g(u_i)$ becomes $g(u_i) = (1+\Delta_{ii})^{a-1}g(s_i) \approx (1+(a-1)\Delta_{ii})g(s_i) = D_{ii}\ g(s_i)$, where D is diagonal matrix with $D_{ii} \equiv 1 + (a-1)\Delta_{ii}$. Using this, we have

$$\begin{aligned}
&\langle\textstyle\sum_{k,l} (g(u_k)\ dK_{kl}\ s_l)\ g(u_i)\ s_j\rangle \approx D_{ii}\ \langle\sum_{k,l} (D_{kk}\ g(s_k)\ dK_{kl}\ s_l)\ g(s_i)\ s_j\rangle \\
&\quad = D_{ii}\ \langle\{\textstyle\sum_l (D_{ii}\ g'(s_i)\ dK_{il}\ s_l) + \sum_k (D_{kk}\ g(s_k)\ dK_{ki})\}\ s_j + \delta_{ij} \sum_{k,l} (D_{kk}\ g(s_k)\ dK_{kl}\ s_l)\rangle \\
&\quad = D_{ii}\ \{\ D_{ii}\ \langle g'(s_i)s_j^2\rangle\ dK_{ij} + D_{jj}\ dK_{ji}\ \Theta[j\le N] + \delta_{ij} \textstyle\sum_k D_{kk}\ dK_{kk}\ \} \\
&\quad = D_{ii}^2\ \langle g'(s_i)s_j^2\rangle\ dK_{ij} + D_{ii}\ D_{jj}\ dK_{ji}\ \Theta[j\le N] + D_{ii}\ \delta_{ij}\ \mathrm{tr}(DdK_1). \qquad \text{(S30)}
\end{aligned}$$

Moreover, by defining $e(u_i) \equiv -\log p_0(u_i) + \langle\log p_0(u_i)\rangle$, we have

$$\begin{aligned}
&\langle(E(\mathbf{u})-E_0)\ g'(u_i)\ dK_{ij}\ s_j^2\rangle = \langle(e(u_i) + (1-\delta_{ij})e(u_j)\Theta[j\le N] - 1)\ g'(u_i)\ s_j^2\rangle\ dK_{ij} \\
&\quad \approx \langle\{(1+a\Delta_{ii})e(s_i) + (1-\delta_{ij})(1+a\Delta_{jj})e(s_j)\Theta[j\le N] - 1\}\ (1+(a-2)\Delta_{ii})g'(s_i)\ s_j^2\rangle\ dK_{ij} \\
&\quad \approx \langle\{(D_{ii}+\Delta_{ii})e(s_i) + (1-\delta_{ij})(D_{ii}+\Delta_{ii}-a\Delta_{ii}+a\Delta_{jj})e(s_j)\Theta[j\le N] - 1\}(D_{ii}-\Delta_{ii})g'(s_i)s_j^2\rangle\ dK_{ij}
\end{aligned}$$

$$= \{(D_{ii}+\Delta_{ii})(D_{ii}-\Delta_{ii})\Omega_{ij} + (D_{ii}-\Delta_{ii})\langle\{(1-\delta_{ij})a(\Delta_{jj}-\Delta_{ii})e(s_j)\Theta[j\le N] - 1\}g'(s_i)s_j^2\rangle\}\ dK_{ij}$$
$$\approx \{D_{ii}^2\ \Omega_{ij} + \langle\{(1-\delta_{ij})a(\Delta_{jj}-\Delta_{ii})e(s_j)\Theta[j\le N] - (D_{ii}-\Delta_{ii})\}g'(s_i)s_j^2\rangle\}\ dK_{ij}$$
$$= \{D_{ii}^2\ \Omega_{ij} + a(\Delta_{jj}-\Delta_{ii})\langle g'(s_i)\rangle\langle e(s_j)s_j^2\rangle\Theta[j\le N] - (D_{ii}-\Delta_{ii})\langle g'(s_i)s_j^2\rangle\}\ dK_{ij}$$
$$= \{D_{ii}^2\ \Omega_{ij} + 2(\Delta_{jj}-\Delta_{ii})\langle g'(s_i)\rangle\Theta[j\le N] - (D_{ii}-\Delta_{ii})\langle g'(s_i)s_j^2\rangle\}\ dK_{ij}. \qquad \text{(S31)}$$

In the last line, $\langle e(s_j)s_j^2\rangle = \langle(g(s_j)s_j - \langle g(s_j)s_j\rangle)s_j^2\rangle/a = 2/a$ is used. Thus, by defining $dX \equiv DdK$, the first term of Eq.(S21) becomes

$$\alpha\ \mathrm{tr}[\langle(\mathbf{s}^T dK^T g(\mathbf{u}) - \langle\mathbf{s}^T dK^T g(\mathbf{u})\rangle)g(\mathbf{u})\mathbf{s}^T + (E(\mathbf{u})-E_0)\ \mathrm{Diag}[g'(\mathbf{u})]\ dK\ \mathbf{s}\mathbf{s}^T\rangle dK^T]$$
$$= \alpha\sum_{i=1}^{N}\sum_{j=1}^{M}[\,D_{ii}^2\ \langle g'(s_i)s_j^2\rangle\ dK_{ij} + D_{ii}\ D_{jj}\ dK_{ji}\ \Theta[j\le N]$$
$$+\ \{D_{ii}^2\ \Omega_{ij} + 2(\Delta_{jj}-\Delta_{ii})\langle g'(s_i)\rangle\Theta[j\le N] - (D_{ii}-\Delta_{ii})\langle g'(s_i)s_j^2\rangle\}\ dK_{ij}]\ dK_{ij}$$
$$= \alpha[\ \sum_{i=1}^{N}\sum_{j=1}^{M}\{\,(D_{ii}^2-D_{ii}+\Delta_{ii})\langle g'(s_i)s_j^2\rangle + D_{ii}^2\ \Omega_{ij}\}dK_{ij}^2$$
$$+\ \sum_{i=1}^{N}\sum_{j=1}^{N}\{\,2(\Delta_{jj}-\Delta_{ii})\langle g'(s_i)\rangle dK_{ij}^2 + D_{ii}\ dK_{ij}\ D_{jj}\ dK_{ji}\}\ ]$$
$$\approx \sum_{i=1}^{N}\sum_{j=1}^{M}\{\,a\Delta_{ii}\langle g'(s_i)s_j^2\rangle+\alpha\Omega_{ij}\}dX_{ij}^2 + \sum_{i=1}^{N}\sum_{j=1}^{N}\{\,2(\Delta_{jj}-\Delta_{ii})\langle g'(s_i)\rangle dX_{ij}^2+\alpha dX_{ij}dX_{ji}\}. \qquad \text{(S32)}$$

In the last line, $D_{ii}^{-2}(D_{ii}^2-D_{ii}+\Delta_{ii}) \approx 1 - D_{ii}^{-1} + \Delta_{ii} \approx 1 - (1-(a-1)\Delta_{ii}) + \Delta_{ii} = a\Delta_{ii}$ is used. In addition, the second and third terms of Eq.(21) become

$$\beta\{\mathrm{tr}[(\Phi\circ(dK^T(I,O)))dK^T(I,O)] + \mathrm{tr}[(\Phi\circ((I,O)^T dK))dK^T(I,O)]\}$$
$$+\ \mathrm{tr}[(\beta\Phi\circ((I,O)^T(I,O) - \Lambda) + \gamma\Lambda^{-1})dK^T dK]$$

$$= \beta\{\mathrm{tr}[(\Phi_1\circ dK_1^T)dK_1^T] + \mathrm{tr}[(\Phi_1\circ dK_1,dK_2)(dK_1,dK_2)^T]\}$$
$$+\ \mathrm{tr}[(\beta\Phi_1\circ(I-\Lambda_1) + \gamma\Lambda_1^{-1})dK_1^T dK_1] + \mathrm{tr}[(\beta\Phi_2\circ(-\Lambda_2) + \gamma\Lambda_2^{-1})dK_2^T dK_2]$$

$$= \beta\{\mathrm{tr}[dK_1(\Phi_1\circ dK_1)] + \mathrm{tr}[dK_1(\Phi_1\circ dK_1^T) + dK_2 dK_2^T]\}$$
$$+\ \mathrm{tr}[(\beta\Phi_1\circ(I-\Lambda_1) + \gamma\Lambda_1^{-1})dK_1^T dK_1] + \mathrm{tr}[(\beta\Phi_2\circ(-\Lambda_2) + \gamma\Lambda_2^{-1})dK_2^T dK_2]$$

$$= \beta\mathrm{tr}[dK_1(\Phi_1\circ(dK_1+dK_1^T))] + \mathrm{tr}[(\beta\Phi_1\circ(I-\Lambda_1) + \gamma\Lambda_1^{-1})dK_1^T dK_1]$$
$$+\ \mathrm{tr}[(\beta(I-\Phi_2\circ\Lambda_2) + \gamma\Lambda_2^{-1})dK_2^T dK_2]$$
$$= \beta\sum_{i=1}^{N}\sum_{j=1}^{N}\ \Phi_{ij}(dK_{ij}+dK_{ji})dK_{ij} + \sum_{i=1}^{N}\sum_{j=1}^{N}(\,\beta\Phi_{jj}(1-\Lambda_{jj})+\gamma\Lambda_{jj}^{-1})dK_{ij}^2$$

$$+\ \sum_{i=1}^{N}\sum_{j=N+1}^{M}(\beta(1-\Phi_{jj}\Lambda_{jj})+\gamma\Lambda_{jj}^{-1})dK_{ij}^{2}. \qquad \text{(S33)}$$

Since $\Delta_1 = (\mathbf{1}\mathbf{1}^T+\Omega_1)^{\dagger}\circ\{\beta\Phi_1\circ(\Lambda_1 - I) - \gamma\Lambda_1^{-1}\} = \mathrm{Diag}[(1+\Omega_{ii})^{-1}(\beta\,\Phi_{ii}(\Lambda_{ii}-1) - \gamma\Lambda_{ii}^{-1})] = \mathrm{Diag}[(\beta\,\Phi_{ii}(\Lambda_{ii}-1) - \gamma\Lambda_{ii}^{-1})\,/\,a]$, Eq.(S33) becomes

$$\beta\sum_{i=1}^{N}\sum_{j=1}^{N}\Phi_{ij}(dK_{ij}+dK_{ji})dK_{ij} - \sum_{i=1}^{N}\sum_{j=1}^{N}a\Delta_{jj}\,dK_{ij}^{2} + \sum_{i=1}^{N}\sum_{j=N+1}^{M}(\beta(1-\Phi_{jj}\Lambda_{jj})+\gamma\Lambda_{jj}^{-1})dK_{ij}^{2}$$

$$\approx\sum_{i=1}^{N}\sum_{j=1}^{N}\{(\beta\Phi_{ij} - a\Delta_{jj})\,dX_{ij}^{2} + \beta\Phi_{ij}\,dX_{ji}\,dX_{ij}\} + \sum_{i=1}^{N}\sum_{j=N+1}^{M}(\beta(1-\Phi_{jj}\Lambda_{jj})+\gamma\Lambda_{jj}^{-1})dX_{ij}^{2}. \qquad \text{(S34)}$$

Therefore, Eq.(S21) becomes

$$d^2L \approx \sum_{i=1}^{N}\sum_{j=1}^{M}\{a\Delta_{ii}\langle g'(s_i)s_j^2\rangle + \alpha\,\Omega_{ij}\}dX_{ij}^{2} + \sum_{i=1}^{N}\sum_{j=1}^{N}\{2(\Delta_{jj}-\Delta_{ii})\langle g'(s_i)\rangle dX_{ij}^{2} + \alpha dX_{ij}dX_{ji}\}$$

$$+\sum_{i=1}^{N}\sum_{j=1}^{N}\{(\beta\Phi_{ij} - a\Delta_{jj})\,dX_{ij}^{2} + \beta\Phi_{ij}\,dX_{ji}\,dX_{ij}\} + \sum_{i=1}^{N}\sum_{j=N+1}^{M}\{\beta(1-\Phi_{jj}\Lambda_{jj})+\gamma\Lambda_{jj}^{-1}\}dK_{ij}^{2}$$

$$\approx\sum_{i=1}^{N}\sum_{j=1}^{N}\{a\Delta_{ii}\langle g'(s_i)s_j^2\rangle+\alpha\Omega_{ij}+2(\Delta_{jj}-\Delta_{ii})\langle g'(s_i)\rangle+\beta\Phi_{ij}-a\Delta_{jj}\}dX_{ij}^{2} + \sum_{i=1}^{N}\sum_{j=1}^{N}(\alpha+\beta\Phi_{ij})dX_{ij}dX_{ji}$$

$$+\ \sum_{i=1}^{N}\sum_{j=N+1}^{M}\{a\Delta_{ii}\langle g'(s_i)s_j^2\rangle + \alpha\Omega_{ij}\}dX_{ij}^{2} + \sum_{i=1}^{N}\sum_{j=N+1}^{M}\{\beta(1-\Phi_{jj}\Lambda_{jj})+\gamma\Lambda_{jj}^{-1}\}dX_{ij}^{2}$$

$$=\sum_{i=1}^{N}\sum_{j=1}^{N}\{a(\Delta_{ii}\langle g'(s_i)s_j^2\rangle-\Delta_{jj})+\alpha\Omega_{ij}+2(\Delta_{jj}-\Delta_{ii})\langle g'(s_i)\rangle+\beta\Phi_{ij}\}dX_{ij}^{2} + \sum_{i=1}^{N}\sum_{j=1}^{N}(\alpha+\beta\Phi_{ij})dX_{ij}dX_{ji}$$

$$+\sum_{i=1}^{N}\sum_{j=N+1}^{M}\{(\beta\Phi_{ii}(\Lambda_{ii}-1)-\gamma\Lambda_{ii}^{-1}+\alpha\frac{a-2}{a})\langle g'(s_i)\rangle + \beta(1-\Phi_{jj}\Lambda_{jj})+\gamma\Lambda_{jj}^{-1}\}dX_{ij}^{2}. \qquad \text{(S35)}$$

Therefore, necessary and sufficient conditions to be linearly stable are given by

For $1 \le i = j \le N$,

$$a(\Delta_{ii}(a-1)-\Delta_{ii}) + \alpha(a-1) + \alpha + 2\beta\Phi_{ii} = a\Delta_{ii}(a-2) + \alpha a + 2\beta\Phi_{ii} > 0$$

For $1 \le i \ne j \le N$,

$$\{a(\Delta_{ii}\langle g'(s_i)\rangle-\Delta_{jj}) + \alpha\langle g'(s_i)\rangle + 2(\Delta_{jj}-\Delta_{ii})\langle g'(s_i)\rangle + \beta\}$$

$$\cdot\ \{a(\Delta_{jj}\langle g'(s_j)\rangle-\Delta_{ii}) + \alpha\langle g'(s_j)\rangle + 2(\Delta_{ii}-\Delta_{jj})\langle g'(s_j)\rangle + \beta\} > \alpha+\beta$$

$$\Longleftrightarrow\quad \alpha^2\,\langle g'(s_i)\rangle\,\langle g'(s_j)\rangle + \langle g'(s_i)\rangle\,\{a(\Delta_{jj}\langle g'(s_j)\rangle-\Delta_{ii}) + 2(\Delta_{ii}-\Delta_{jj})\langle g'(s_j)\rangle + \beta\}$$

$$+\ \langle g'(s_j)\rangle\,\{a(\Delta_{ii}\langle g'(s_i)\rangle-\Delta_{jj}) + 2(\Delta_{jj}-\Delta_{ii})\langle g'(s_i)\rangle + \beta\} > 1$$

$$\Longleftrightarrow\quad (1-2\beta)\langle g'(s_i)\rangle^2 + a\langle g'(s_i)\rangle(\langle g'(s_i)\rangle-1)(\Delta_{ii}+\Delta_{jj}) + 2\beta\langle g'(s_i)\rangle > 1$$

$$\Longleftrightarrow\quad (\langle g'(s_i)\rangle+1)(\langle g'(s_i)\rangle-1) - 2\beta\langle g'(s_i)\rangle(\langle g'(s_i)\rangle-1) + a\langle g'(s_i)\rangle(\langle g'(s_i)\rangle-1)(\Delta_{ii}+\Delta_{jj}) > 0$$

$$\Longleftrightarrow \quad (\langle g'(s_i)\rangle - 1)\{\langle g'(s_i)\rangle + 1 - 2\beta\langle g'(s_i)\rangle + a\langle g'(s_i)\rangle(\Delta_{ii}+\Delta_{jj})\} > 0,$$

For $1 \le i \le N$, $N+1 \le j \le M$,

$$(\beta\Phi_{ii}(\Lambda_{ii}-1) - \gamma\Lambda_{ii}^{-1} + \alpha\frac{a-2}{a})\langle g'(s_i)\rangle + \beta(1 - \Phi_{jj}\Lambda_{jj}) + \gamma\Lambda_{jj}^{-1} > 0. \tag{S36}$$

The first and second inequalities are satisfied when $p_0(s_i) = \exp(-b|s_i|^a)/Z$. Thus, the linear stability depends on the third inequality.

S4. Conventional PCA algorithm

Oja's subspace rule [11] is defined by

$$\begin{aligned} \dot{W} &\propto \langle \mathbf{u}(\mathbf{x}^T - \mathbf{u}^T W)\rangle \\ &= K\langle \mathbf{s}\mathbf{s}^T\rangle A^T - K\langle \mathbf{s}\mathbf{s}^T\rangle K^T W. \end{aligned} \tag{S37}$$

When $\langle \mathbf{s}\mathbf{s}^T\rangle = I$, it becomes

$$\dot{W} \propto K(A^T A - K^T K)A^{-1}. \tag{S38}$$

This is equivalent to the EGHR-β with (α, β, γ) = (0, 1, 0) except positive definite matrix AA^T and kurtosis terms.
